# Supplementary material for: Analysis of the potential for point-of-care test to enable individualised treatment of infections caused by antimicrobial-resistant and susceptible strains of Neisseria gonorrhoeae: a modelling study
Source: BMJ Open. 2017 Jun 14;7(6):e015447. doi: 10.1136/bmjopen-2016-015447 (PMC5734280; doi:10.1136/bmjopen-2016-015447)
Supplement: Supplementary appendix [file bmjopen-2016-015447supp001.pdf]

Supplementary information for:

Analysis of the potential for point-of-care test to enable individualised treatment of infections caused by antimicrobial-resistant and susceptible strains of *Neisseria gonorrhoeae*

Katy ME Turner, Hannah Christensen, Elisabeth J Adams, David McAdams, Helen Fifer, Anthony McDonnell, Neil Woodford

Table A1 Current prevalence of antimicrobial resistance to potential treatments for gonorrhoea

Drug	Class	Prevalence of resistance in GRASP 2014 isolates[1]			
		Heterosexual men	MSM	Women	Overall
Ceftriaxone	Cephalosporin (3rd generation)	0	0	0	0
Penicillin	β-lactam	18%	26%	10%	23%
Ciprofloxacin	Fluoroquinolone	28%	44%	15%	37%
Azithromycin	Macrolide	0.0%	1.4%	0.5%	1.0%

Current first line (and last-line) therapy is intramuscular ceftriaxone with azithromycin 1g orally. MSM, men who have sex with men.
GRASP: Gonococcal Resistance to Antimicrobial Surveillance Programme

Table A2 Model parameters

Baseline model parameters	Current			AMR POCT		
	Heterosexual men	MSM	Women	Heterosexual men	MSM	Women
Initial population size[2]	515,094	145,863	779,085	515,094	145,863	779,085
Proportion entering same day management pathway	35%	33%	48%	100%	100%	100%
Proportion infected with gonorrhoea (of total tested)[2]	1.5%	12.4%	1.1%	1.5%	12.4%	1.1%
Proportion of those in same day pathway infected with gonorrhoea	3.1%	26.0%	1.0%	1.5%	12.4%	1.1%
Proportion of delayed management infected with gonorrhoea	0.7%	5.6%	1.2%	-	-	-
Relative risk infection gonorrhoea in same day vs delayed pathway	4.52	4.63	0.82	-	-	-
Proportion in same day pathway who are infected & treated on same day	96%	90%	50%	100%	100%	100%
Proportion of same day pathway treated presumptively for gonorrhoea	5.0%	25.0%	2.0%	1.5%	12.4%	1.1%
Proportion who attend for treatment after lab test result (of those who wait for lab test results, i.e. asymptomatic group)	95%	95%	95%	100%	100%	100%
Proportion treated with last line therapy[3]	100%	100%	100%	28%[a]	44%[a]	15%[a]
Cost of first attendance[4,5]	£135	£135	£135	£135	£135	£135
Cost of follow-up attendance[4,5]	£104	£104	£104	£104	£104	£104

AMR POCT, antimicrobial resistance point of care test; MSM, men who have sex with men.

[a]Assuming POCT for ciprofloxacin susceptibility (can be adjusted for penicillin according to parameters in Table A1 or updated to reflect local trends)

Figure A1

A – Heterosexual men current pathway

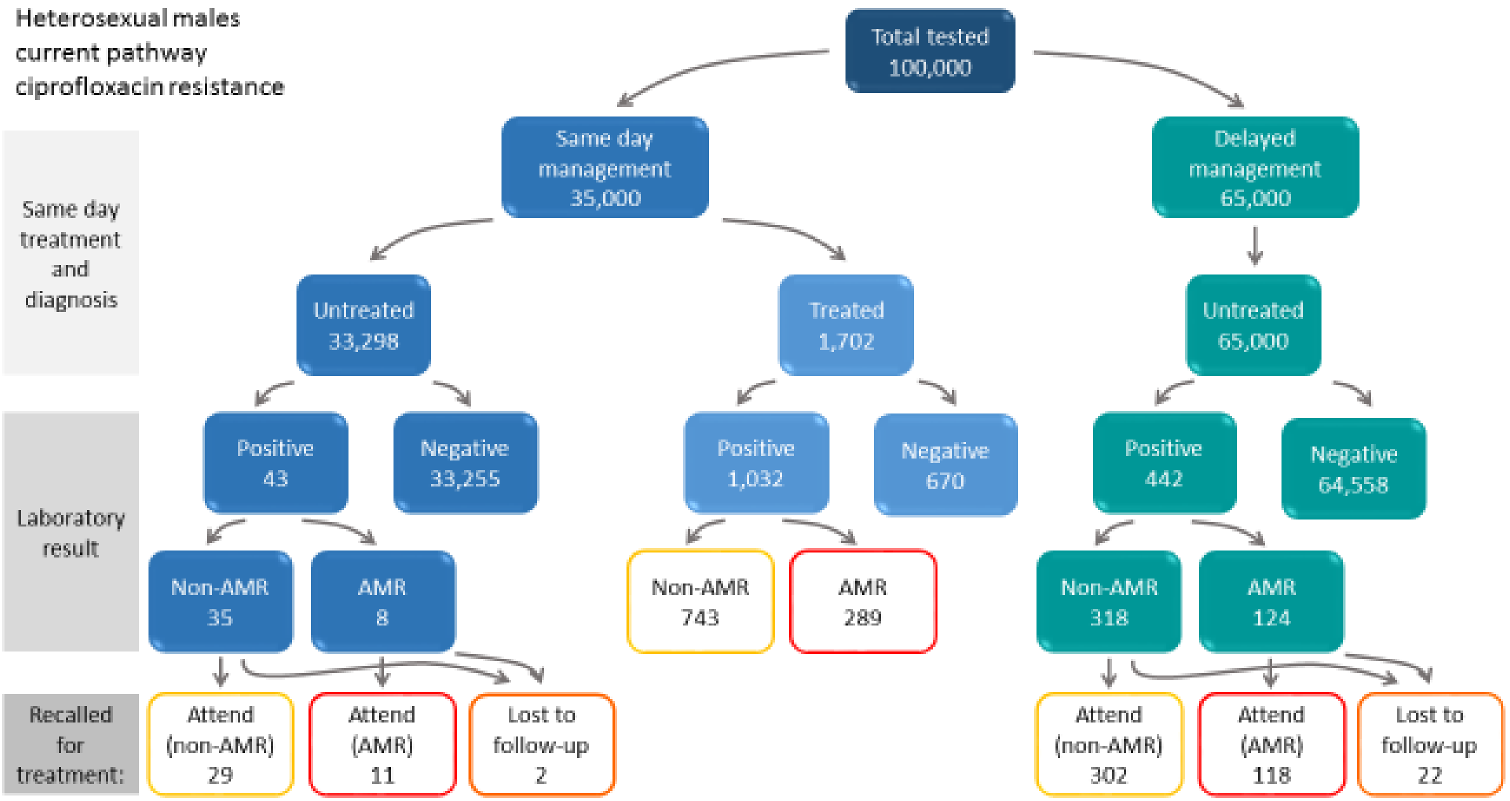

B – Heterosexual men POCT pathway

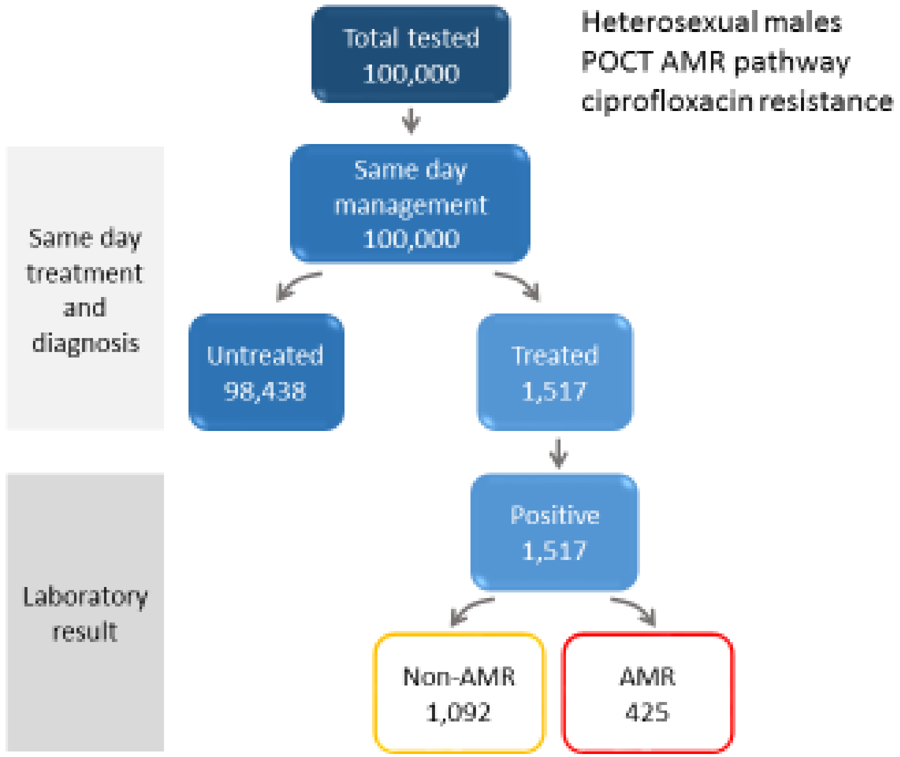

C – Women current pathway

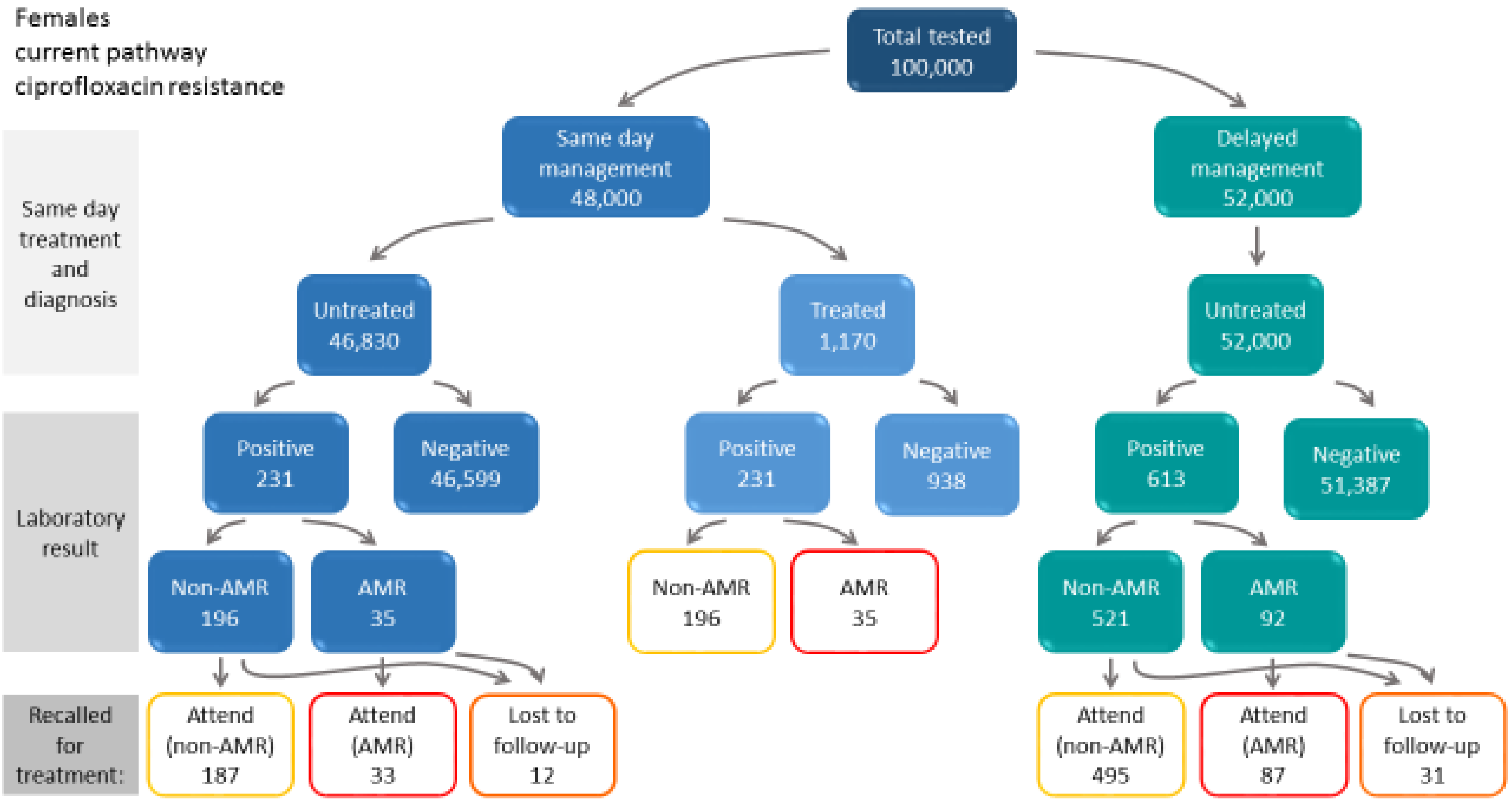

D – Women new pathway

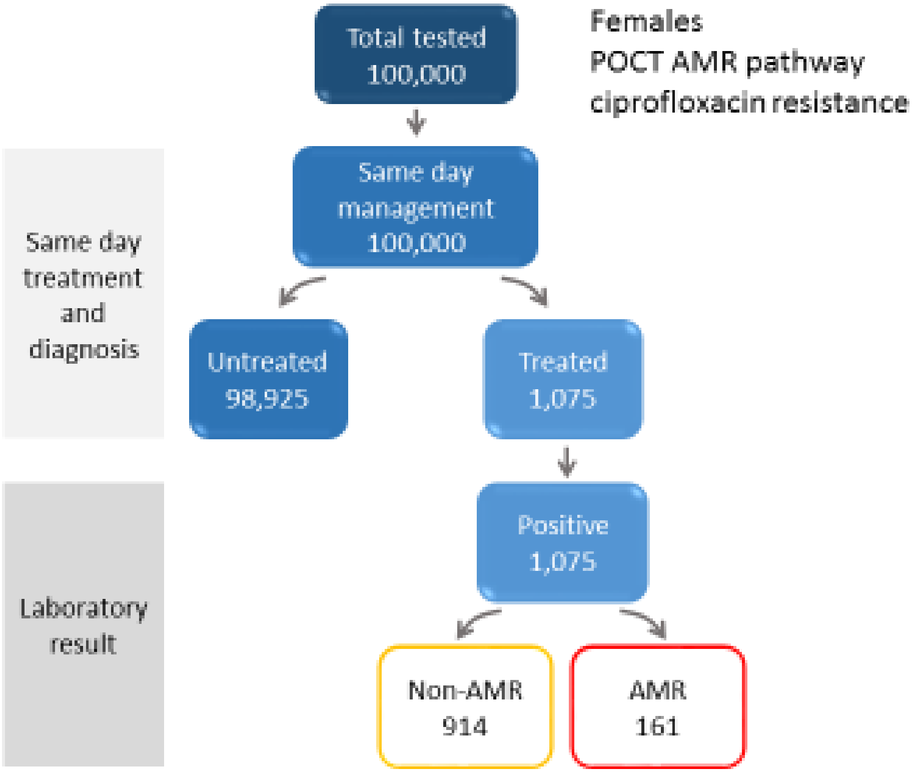

References

1 Adams EJ, Ehrlich A, Turner KM, Shah K, Macleod J, Goldenberg S, et al. Mapping patient pathways and estimating resource use for point of care versus standard testing and treatment of chlamydia and gonorrhoea in genitourinary medicine clinics in the UK. BMJ Open 2014; 4(7): e005322.

2 Public Health England. STI diagnoses & rates by gender, sexual risk & age group, 2010 - 2014, 2015.

3 England. PH. Surveillance of antimicrobial resistance in *Neisseria gonorrhoeae*. Key findings from the 'Gonococcal resistance to antimicrobials surveillance programme' (GRASP) and related surveillance data, 2014.

4 Monitor and NHS England. 2014/15 National Tariff Payment System, 2013.

5 Health PHEatDo. HIV, sexual and reproductive health: current issues bulletin2013. Available from: https://www.gov.uk/government/uploads/system/uploads/attachment_data/file/259087/HIV_Sexual_and_Reproductive_Health_bulletin-issue1nov2013.pdf.

6 Turner KM, Round J, Horner P, Macleod J, Goldenberg S, Deol A, et al. An early evaluation of clinical and economic costs and benefits of implementing point of care NAAT tests for *Chlamydia trachomatis* and *Neisseria gonorrhoea* in genitourinary medicine clinics in England. Sex Transm Infect 2014; 90(2): 104-11.
