# Supplementary material for: Analysis of the potential for point-of-care test to enable individualised treatment of infections caused by antimicrobial-resistant and susceptible strains of Neisseria gonorrhoeae: a modelling study
Source: BMJ Open. 2017 Jun 14;7(6):e015447. doi: 10.1136/bmjopen-2016-015447 (PMC5734280; doi:10.1136/bmjopen-2016-015447)
Supplement: Author's manuscript [file bmjopen-2016-015447.draft_revisions.pdf]

BMJ Open

Analysis of the potential impact of a point-of-care test to distinguish gonorrhoea cases caused by antimicrobial-resistant and susceptible strains of *Neisseria gonorrhoeae*

For peer review only

Journal:	*BMJ Open*
Manuscript ID	bmjopen-2016-015447
Article Type:	Research
Date Submitted by the Author:	05-Dec-2016

Complete List of Authors:	Turner, Katy; Bristol University<br>Christensen, Hannah; University of Bristol, School of Social and Community Medicine<br>Adams, Elisabeth; Aquarius Population Health, Managing Director and Founder<br>McAdams, David ; Duke University, Duke Fuqua School of Business<br>Fifer, Helen; Public Health England Colindale, Bacteriology Reference Department<br>McDonnell, Anthony ; Wellcome Trust<br>Woodford, Neil; Public Health England, National Infection Service

**Primary Subject Heading**:	Infectious diseases
Secondary Subject Heading:	Diagnostics, Epidemiology
Keywords:	INFECTIOUS DISEASES, Diagnostic microbiology < INFECTIOUS DISEASES, HEALTH ECONOMICS

SCHOLARONE™ Manuscripts

Analysis of the potential for point-of-care test to enable individualised treatment of infections caused by antimicrobial-resistant and susceptible strains of *Neisseria gonorrhoeae*

Katy ME Turner, Hannah Christensen, Elisabeth J Adams, David McAdams, Helen Fifer, Anthony McDonnell, Neil Woodford

School of Veterinary Sciences, University of Bristol, Langford House, Langford, Bristol BS40 5DU, UK
Katy ME Turner
Senior Lecturer

School of Social and Community Medicine, University of Bristol, Oakfield House, Oakfield Grove, Bristol, BS8 2BN, UK
Hannah Christensen
Lecturer

Aquarius Population Health, 58a Highgate High Street, London N6 5HX, UK
Managing Director and Founder
Elisabeth Adams

Duke Fuqua School of Business, 100 Fuqua Drive, A416, Durham, NC 27708, USA
Professor of Business Administration and Economics
David McAdams

Bacteriology Reference Department, National Infection Service, Public Health England, London, UK
Consultant Microbiologist
Helen Fifer

The O'Neill Review on Antimicrobial Resistance, Wellcome Trust, London, UK
Head of Economic Research
Anthony McDonnell

Bacteriology Reference Department, National Infection Service, Public Health England, London, UK
Head, AMRHAI Reference Unit
and
The O'Neill Review on Antimicrobial Resistance, Wellcome Trust, London, UK
Scientific Advisor
Neil Woodford

Correspondence to:
Katy ME Turner
E: katy.turner@bristol.ac.uk
T: +44 (0) 117 3314563

Keywords: point-of-care test; Neisseria gonorrhoeae; antimicrobial-resistance

Word count: 2939
Excluding title page, abstract, references, figures and tables.

For peer review only

For peer review only - http://bmjopen.bmj.com/site/about/guidelines.xhtml

Abstract

Objective: To create a mathematical model to investigate the treatment impact and economic implications of introducing an antimicrobial resistance point-of-care test (AMR POCT) for gonorrhoea as a way of extending the life of current last-line treatments.

Design: Modelling study.

Setting: England.

Population: Patients accessing sexual health services.

Interventions: Incremental impact of introducing a hypothetical AMR POCT that could detect susceptibility to previous first line antibiotics e.g. ciprofloxacin or penicillin so that patients are given more tailored treatment, compared with the current situation where all patients are given therapy with ceftriaxone and azithromycin. The hypothetical intervention was assessed using a mathematical model developed in Excel. The model included initial and follow-up attendances, loss to follow-up, use of standard or tailored treatment, time taken to treatment and the costs of testing and treatment.

Main outcome measures: Number of doses of ceftriaxone saved, mean time to most appropriate treatment, mean number of visits per (infected) patient, number of patients lost to follow-up and total cost of testing.

Results: In the current situation an estimated 33,431 ceftriaxone treatments are administered annually and 792 gonococcal infections remain untreated due to loss to follow-up. The use of an AMR POCT for ciprofloxacin could reduce these ceftriaxone treatments by 66%, and for an AMR POCT for penicillin by 79%. The mean time for patients receiving an antibiotic treatment is reduced by 2 days in scenarios including POCT and no positive patients remain untreated through eliminating loss to follow-up. Such POCTs are estimated to add £34 million to testing costs, but this does not take into account reductions in costs of repeat attendances and the reuse of older, cheaper antimicrobials.

Conclusions: The introduction of AMR POCT could allow clinicians to discern between the majority of gonorrhoea-positive patients with strains that could be treated with older, previously abandoned first-line treatments, and those requiring our current last-line dual therapy. Such tests could extend the useful life of dual ceftriaxone and azithromycin therapy, thus pushing back the time when gonorrhoea may become untreatable.

For peer review only - http://bmjopen.bmj.com/site/about/guidelines.xhtml

What is already known on this subject

- The rise of antimicrobial resistant gonorrhoea is a pressing public health problem because gonorrhoea is the second most common bacterial sexually transmitted infection in the UK and untreated disease can lead to serious complications.
- The currently recommended first-line treatment of ceftriaxone and azithromycin is also our last-line treatment option, raising concern that gonorrhoea could soon become untreatable.
- Most strains of *Neisseria gonorrhoeae* in the UK are susceptible to older, abandoned first-line treatments, but characterisation of the resistance/susceptibility profiles of infection is not available at the time of diagnosis and treatment.

What this study adds

- Model estimates suggest the use of a hypothetical antimicrobial resistance point-of-care test (AMR POCT) for ciprofloxacin resistance could prevent 22,054 treatments with ceftriaxone annually (66% reduction), and an AMR POCT for penicillin resistance could prevent 26,499 ceftriaxone treatments (79% reduction).
- The use of AMR POCTs could reduce the mean time for patients receiving the most appropriate treatment by two days and prevent positive individuals remaining untreated through the elimination of loss to follow-up.
- Assuming an AMR POCT added £25 to the testing costs, using such a test would increase the total cost of testing in England by £34 million annually, though a proportion of this would be offset though reductions in (re)testing and the re-use of older cheaper antimicrobials.

For peer review only

For peer review only - http://bmjopen.bmj.com/site/about/guidelines.xhtml

INTRODUCTION

Increasing antimicrobial resistant gonorrhoea represents a significant and urgent public health problem. Gonorrhoea, caused by *Neisseria gonorrhoeae* is the second most commonly diagnosed bacterial sexually transmitted infection (STI) in England. *N. gonorrhoeae* has evolved resistance to all major drug classes and has been recognised as a bacterium of international concern by World Health Organization (WHO) [1] and has been prioritised in the UK five-year antimicrobial resistance strategy[2].

Diagnoses have more than doubled from 16,839 in 2010 to 41,193 in 2015, mainly due to increased diagnoses in men who have sex with men (MSM), accounting for 70% of male infections in 2015, illustrated in Figure 1 [3] (data reported through GUMCADv2, including GUM clinics and other sexual health service providers, but not general practice). Infections are often asymptomatic, especially in women and in pharyngeal and rectal infections in MSM, but are still transmissible[5]. If untreated, complications of infection include pelvic inflammatory disease, infertility, increased risk of pregnancy complications and, in rare cases, life-threatening septicaemia[6]. Gonorrhoea infection also increases the risk of HIV acquisition[7].

[3]In the UK, the Gonococcal Resistance to Antimicrobial Surveillance Program (GRASP) has performed sentinel antibiotic susceptibility testing of gonorrhoea since 2000[11]. Increases in resistance to first line therapies resulted in two changes in treatment recommendation (Figure 1): from ciprofloxacin to cefixime in 2005 and then to ceftriaxone plus azithromycin in 2011 [8,10,11]. Our current first-line therapy is also our last-line option, and whilst the use of dual therapy is intended to delay resistance developing to ceftriaxone, decreased susceptibility to either of these drugs could lead to untreatable infections. Whilst new antibiotics are in development, their use in the clinic may be many years away and already the world's first reported clinical treatment failure with confirmed ceftriaxone and azithromycin resistance has occurred [11].

There are two main challenges to the management of gonorrhoea which contribute to the problem of resistance, illustrated in Figure 2. 1) Precautionary treatment: at the time of diagnosis, such that all infections are treated as if they are resistant to older antibiotics and 2) Epidemiological treatment: sexual contacts of gonorrhoea cases are often treated before diagnostic test results are known resulting in unnecessary treatment of uninfected partners. The cornerstone of gonorrhoea management to date has been to ensure rapid, highly effective treatment is given to prevent the onward spread of infection to sexual partners and

For peer review only - http://bmjopen.bmj.com/site/about/guidelines.xhtml

to prevent people not returning for treatment following a diagnosis. In the context of antibiotic resistance and new diagnostic technologies, it is necessary to reassess these priorities.

Strategies are required to extend the life of existing antimicrobials for the successful treatment of gonorrhoea. Most infections diagnosed in the UK are susceptible to cefixime, ciprofloxacin and even penicillin [11]. Therefore, if a point-of-care test (POCT) could be developed to test for resistance (or susceptibility) to antibiotics, most patients could be treated with an older oral first-line therapy,potentially extending the life of ceftriaxone as our last-line therapy. [12]A promising option based on existing nucleic acid amplification test (NAAT) could be a PCR test for ciprofloxacin resistance, using the gyrA Gene as a target [12,13]. Other technologies could involve direct measurement of live cell responses to the presence of a panel of antibiotics including microfluidic devices, atomic force microscopy, volatile chemical detection or mass spectroscopy. Computational approaches based on *in silico* phenotyping based on genotype may also be able to detect new mutations more rapidly than traditional microbiological testing [14-16]. In this study we developed a mathematical model to investigate the treatment impact and economic implications of introducing an antimicrobial resistance (AMR) POCT for gonorrhoea.

METHODS

Model

We developed a decision tree model in Excel to consider the impact of a hypothetical new AMR POCT on testing, diagnosis and treatment of gonorrhoea in sexual health clinics in England (Figure 3), compared to current practice. Genitourinary clinics typically triage attending patients based on whether they have symptoms or report contact with a sexual partner infected with a specific infection ("same day management") and those without symptoms ("delayed management") where treatment is delayed until the results of diagnostic tests are returned from the laboratory (2-7 days) (Figure 2). Current practice is therefore a mixture of same day management and delayed management depending on clinic patient mix. Guidelines recommend that patients treated for gonorrhoea also have swabs taken at the time of treatment that are sent for susceptibility testing, but these results are not available until after treatment has been given. The alternative strategy is based on a point of care gonorrhoea diagnostic test for all patients. The point of care test (POCT) could be either a simple diagnostic for gonorrhoea (infected/not infected) or a test which can discriminate between one specific resistance/susceptibility determinants (POCT AMR). More complex testing algorithms and diagnostic technologies could be envisioned, for example only using an AMR POCT if the initial simple POCT is positive (reflex testing) or using more complex algorithms and new technologies to determine optimal treatment options. In this preliminary

For peer review only - http://bmjopen.bmj.com/site/about/guidelines.xhtml

example we consider two options of antimicrobial susceptibility 1) ciprofloxacin and 2) penicillin. [17,18]

The model was based upon an existing pathway model used to investigate the impact of introducing a dual POCT for gonorrhoea and chlamydia in a genitourinary medicine (GUM) setting[18,19], but simplified in that onward transmission of gonorrhoea and partner notification were not included, with the focus being on diagnosis and tailored treatment, shown in Figure 3. We explicitly included branches to differentiate susceptible and resistant isolates within the pathway framework. For the purpose of our study, we assumed that all point of care tests are 100% sensitive and specific for simplicity. Previous models have considered variable specificity and sensitivity requirements in more detail [17].

Hypothetical cohorts of patients were followed through the pathway (MSM, heterosexual men and heterosexual women). Individuals could either receive same-day management or delayed management (Figure 3) under current practice or for POCT pathway all patients are assumed tested, diagnosed and treated on the same day. The only difference between POCT and AMR POCT is therefore in the choice of antimicrobial therapy. Treatments modelled were either our current last-line dual therapy of ceftriaxone and azithromycin (current pathway or simple POCT), or in the case of scenarios including AMR POCT a proportion of patients were provided with either ciprofloxacin or penicillin, plus azithromycin co-therapy, as an alternative regimen where possible. Loss to follow-up when patients were recalled for treatment following laboratory testing to determine positivity for gonorrhoea was explicitly included for current pathway only. We assumed that results of point of care diagnostics can be provided within the clinical consultation, e.g. if patients provide samples for testing on arrival at a GUM clinic and then wait for an appointment or return later in the day. It is possible that this would result in delays to treatment for symptomatic individuals and sexual contacts, but we do not consider this further here as we are exploring the potential of theoretical new tests.

Parameter values

Full model parameters are provided in the Appendix Table A1 and Table A2. Estimates of the numbers of patients attending GUM clinics and tested for and diagnosed with gonorrhoea were based on recent data from Public Health England (PHE)[4]. The model is run assuming 515,094 MSW, 145,863 MSM and 779,085 women attend a GUM clinic in 2014)[4] and the proportions entering same day management or who are infected adjusted to generate the observed diagnoses of gonorrhoea in each group. In 2014, there were over 33,000 diagnoses of gonorrhoea reported by PHE, just over half in men who have sex with

For peer review only - http://bmjopen.bmj.com/site/about/guidelines.xhtml

men (MSM) and the remaining heterosexual cases split roughly equally between men and women. We combined data on patients presenting as contacts of gonorrhoea cases or with symptoms into the "same-day management" pathway. Asymptomatic patients were tested, but treatment was assumed to be delayed until the results of laboratory tests were known. We distributed infected patients between the pathways according to specific parameters for each patient group based on the probability of being infected and the likelihood of having symptoms. Symptomatic patients are more likely to be managed on the same day as testing and heterosexual men (MSW) are the most likely to be symptomatic, followed by MSM, then women. (Data from the Maximising STI Control trial, personal communication Cath Mercer) (Table 1) [18,19,20]. These parameters were informed by national PHE data where available and supplemented with additional data or clinical experience and are described fully elsewhere [18,20]. The difference between MSM and MSW may be due to a combination of factors including higher probability of extra-genital infection, higher incidence of repeat infections and higher probability of HIV coinfection and higher frequency of STI testing in this group.[21] We estimated the proportions of infections that are resistant to ciprofloxacin and/or penicillin from the GRASP 2014 report, which included systematic susceptibility testing at the PHE reference laboratory from sentinel surveillance sites and a larger but less well defined analysis of samples tested locally[9]. Parameters were varied to be appropriate to three patient groups: heterosexual men, MSM, and women. In the baseline case we assumed that all confirmed and presumptive gonorrhoea infections are treated with ceftriaxone and azithromycin because there is >5% resistance to alternative regimens, resulting in 100% of infections treated as if they are resistant to other antibiotics (such as ciprofloxacin). The cost for patients attending GUM were taken from the latest payment by results tariff[22]. An AMR POCT is not currently available so we assumed conservatively that separate new tests for assessing resistance to either ciprofloxacin or penicillin would each incur an additional £25 testing cost.

Management scenarios

We considered the following scenarios for each of the three patient groups (MSM, heterosexual men and women).

1) Current management – clinicians have no knowledge of the resistance profile of gonorrhoea at the point of initial treatment and consequently all patients are treated with ceftriaxone and azithromycin. Some patients are managed on the same day, either due to symptoms and positive microscopy or as contacts of infected individuals, others wait for lab results, resulting in some unnecessary treatment and some delays to treatment or loss to follow-up. (Figure 3A)

For peer review only - http://bmjopen.bmj.com/site/about/guidelines.xhtml

2) Simple POCT management – all patients tested and managed same day but all treated as if resistant to older antibiotics (i.e. ceftriaxone and azithromycin)
3) AMR POCT management - all patients tested with AMR POCT for gonorrhoea that could identify infections that do not need to be treated with ceftriaxone
    a. assuming current ciprofloxacin resistance prevalence[9]. (Figure 3B)
    b. assuming current penicillin resistance prevalence[9].

Economic analysis

The primary outcomes were: the number of doses of ceftriaxone saved; and the mean time to appropriate treatment. In addition, we calculated the average number of visits per person and per infected person, the total cost of testing and the number of patients lost to follow up. In each case we compared the incremental benefit of an AMR POCT with current testing practice. Analyses were undertaken from the NHS perspective with costs measured in pounds sterling at 2014 prices.

RESULTS

We modelled a snapshot of GUM attendance, gonorrhoea diagnosis and prevalence of resistance to ciprofloxacin and penicillin based on the situation in England, 2014[4]. Under current treatment guidelines for 1.4 million people attending GUM per year we estimate 33,431 ceftriaxone treatments are currently administered annually and 792 gonococcal infections remain untreated due to loss to follow-up. In those receiving antibiotics, the mean time to treatment was estimated to be 2.2 days. Under current practice, 68% (MSW), 63% (MSM) and 21% (Women) who are infected with gonorrhoea are treated on the same day as they attend. The mean number of attendances at clinic per infected person was 1.44. We estimated the total cost of current testing to be £196 million. If a POCT test is used (strategies 2-4), this enables same-day testing and treatment, patients would only need to visit once, all infected individuals would be treated on the same day as the test and therefore no infected individuals would be lost to follow-up and left untreated.

The results for AMR POCT (strategy 3, 4) and POCT (strategy 2) only differ by the choice of treatment regimen. If an AMR POCT for ciprofloxacin resistance were available (strategy 3a) we estimate its use could prevent 22,054 treatments of ceftriaxone annually (a 66% reduction) assuming the current levels of resistance to ciprofloxacin (37% of infections in 2014[23],

For peer review only - http://bmjopen.bmj.com/site/about/guidelines.xhtml

Table 1). Similarly an AMR POCT for penicillin resistance (strategy 3b) at the current levels of resistance (23% overall) could prevent 26,499 ceftriaxone treatments annually (a 79% reduction). Assuming an AMR POCT added £25 to the testing costs we estimated the total cost of testing for each of the POCT scenarios to be £230 million, adding £34 million to the annual cost of testing.

DISCUSSION

Statement of principal findings

Our model estimates that 66% of the 33,431 ceftriaxone treatments given annually to individuals with gonorrhoea could be replaced by ciprofloxacin, thus extending the life of our current last-line treatment, if an AMR POCT for ciprofloxacin resistance was available. If an AMR POCT for penicillin was available, 79% of ceftriaxone treatments could be substituted with penicillin. The use of POCTs would mean a two day reduction in the time that people wait, on average, for appropriate treatment compared with current practice and such testing would prevent the approximately 800 positive individuals who remain untreated in the current system due to loss to follow-up. If AMR POCT added £25 to first-line testing costs, we estimate the use of such tests would increase current treatment and testing costs by £34 million annually. The outcomes related to same day diagnosis and treatment (reduced time to treatment and reduced follow up) could be achieved by using a simple POCT, as previously considered[18]. The additional benefit of AMR POCT test is to enable tailored choice of antimicrobial treatment.

Strengths and weaknesses of the study

Our model used recent published data on antimicrobial resistance levels, gonococcal incidence and current treatment and considered the impact of additional AMR POCT in distinct population groups, namely heterosexual men, MSM and females. The simplified model structure, which is available freely online, enables the parameters to be easily updated and the impact of different scenarios, in different settings, to be considered. We made the simplifying assumption that the cost of an AMR POCT would add £25 to the current tariff cost; however, in reality other current activities might be reduced or discontinued if an AMR POCT was available, such as testing, microscopy, culture and physical exams or re-attendances, as well as reduced costs associated with re-using cheaper oral antibiotics. New DNA-based POCT technologies may be able to be combined to produce a multiplexed test, which may be more economically viable than the separate specific AMR tests we modelled here. Our cost estimates are therefore likely to be higher than in practice. New technologies are emerging which may be able to rapidly determine the bacterial response to a panel of potential antibiotics which would enable highly tailored

For peer review only - http://bmjopen.bmj.com/site/about/guidelines.xhtml

therapy without the need to continuously monitor the efficacy of a test for resistance based on detecting DNA sequence, but for this preliminary exploration we selected a hypothetical AMR POCT test which could integrate with existing POCT technologies based on nucleic acid amplification.

The model did not capture the indirect effects of reduced transmission to partners or progression to complications, such as pelvic inflammatory disease and epididymitis. It also did not consider the longer term effects of changing treatment strategy on the evolution of drug resistance over time in gonorrhoea infections.

Strengths and weaknesses in relation to other studies, discussing important differences in results

To our knowledge, no-one has specifically addressed the question of the added value of a point-of-care AMR POCT to discriminate between susceptible and resistant strains to guide initial treatment decisions for gonorrhoea. Others have considered in detail the relative benefits of POCTs, balancing the need for fast results against cost and test performance[17]. Adams et al previously showed that a dual chlamydia/gonorrhoea point of care NAAT diagnostic test pathway could be cost neutral or cost-saving compared with existing methods even though the test kit itself is more expensive. [18,19] We initially assumed that the POCT AMR is an additional test cost, however it is probable that a multiplex PCR rapid test could be designed to include an AMR component which does not compromise the cost or performance of the basic gonorrhoea diagnostic. An alternative to improving diagnostics, treatment and surveillance is to develop a vaccine for gonorrhoea and to improve the uptake of other methods of prevention (such as condoms)[24,25]. A gonorrhoea vaccine has proved elusive due to the rapidly changing surface antigens, but there may be some cross-reactivity with vaccines designed to protect against *Neisseria meningitidis*[26].

The main weakness of our study is that it did not address the population level impact of the introduction of such tests, but only considered a static situation[23,24,27]. Rapid whole genome sequencing (within 24 hours) has been introduced to help guide treatment decisions for important nosocomial pathogens, notably MRSA (methicillin-resistant *Staphylococcus aureus*)[16], but in a community walk-in clinic setting for a low prevalence bacterial infection, such as gonorrhoea, a test needs to be relatively cheap and results available before the patient leaves the clinic . Our model did not include dynamic epidemiological or evolutionary processes, which change the prevalence and incidence of infection (and resistance) over time[23]. In reality, re-introduction of ciprofloxacin would likely increase the selection for resistance, which would negate some of the benefits of an AMR POCT. Similarly re-using

For peer review only - http://bmjopen.bmj.com/site/about/guidelines.xhtml

other drugs would also result in increases in resistance observed, including increasing selection for plasmids conferring multidrug resistance. Conversely, if point-of-care technology can reduce the time to treatment and reduce loss to follow-up sufficiently this might reduce the overall population prevalence, which would lead to a virtuous cycle of improved control and reduced transmission risk[28]. We also assume that results of point of care diagnostics can be provided within the clinical consultation. This is not currently possible unless the patient provides samples on arrival then waits to see a clinician or returns for a later appointment. The Cepheid GeneXpert has a turnaround time of about 90 minutes which was previously found to result in the majority of men (16/19) not waiting for their results (6 were positive)[30]. Transmission dynamic models can explore the potential consequences without the risks associated with radical changes in prescribing practices. The next steps will be to develop dynamic models which include selective pressure under differing treatment options[29] and incorporating variable delays.

The important next questions arising from this study are: how much time does the reduction in use of ceftriaxone buy in terms of slowing or preventing the emergence of clinically relevant gonorrhoea resistant to ceftriaxone and, second, what are the population-level benefits of improved gonorrhoea control?

Meaning of the study: possible explanations and implications for clinicians and policymakers

The major benefit of point of care tests for gonorrhoea is increasing the proportion of patients treated appropriately on the same day as the test, which is likely to improve outcomes by reducing infectious duration, reducing loss to follow-up and potentially improving partner notification efficacy. A definitive diagnosis on the day of first presentation also prevents unnecessary treatment of those not infected with gonorrhoea. The main benefit of an AMR POCT that can discriminate between susceptible and resistant infections is in enabling the re-introduction of abandoned first-line therapies. Reducing the use of antibiotics, especially of last-line therapies is a key aim of the UK national strategy on antimicrobial resistance. For heterosexual men and MSM a relatively large proportion of infections are already treated on the same day as testing, based on epidemiological, clinical or microbiological evidence (microscopy). However, this proportion is lower for women due to the higher percentage of asymptomatic infections and from poorer sensitivity of detection of gonorrhoea in endocervical and urethral smears. Although new POCTs are likely to be

For peer review only - http://bmjopen.bmj.com/site/about/guidelines.xhtml

more expensive than existing tests this would to some extent be offset by the reduction in further attendances and in the ability to re-use older, cheaper drugs. Given the low prevalence of gonorrhoea even in high-risk GUM attendees, the cost of treatment and re-attendances is small in comparison with the cost of attendances for testing and diagnosis. If a new discriminatory AMR POCT test were prohibitively expensive for routine use, a combination of a standard point-of-care NAAT (e.g. chlamydia/gonorrhoea) test could be considered in conjunction with a more specialised gonorrhoea AMR test, although the time implications of this for patients and clinicians would have to be carefully considered.

Unanswered questions and future research

This estimation of the potential reduction in ceftriaxone use is the first step towards evaluating the long-term effects of such a reduction. Future research investigating how much the useful lifespan of ceftriaxone as a therapy for gonorrhoea is extended with particular reductions in ceftriaxone use would be valuable. In the context of the often slow and expensive new drug pipeline, there is also a question to be answered around the value placed on each additional year of ceftriaxone availability.

For peer review only

For peer review only - http://bmjopen.bmj.com/site/about/guidelines.xhtml

Acknowledgments

We would like to acknowledge Lord Jim O'Neill, Chairman of the Review on Antimicrobial Resistance and the review team for commissioning the study.

Contributors

All authors were involved in the conception and design of the research. KT, EA and HC developed the models, following initial work by DM and NW and based on previous published work by EA & KT; KT and HC analysed the model results and all authors interpreted the results. HF and NW provided input into current clinical practice relating to AMR. KT, HC and NW wrote the first draft of the manuscript; all authors drafted the final version of the manuscript. All authors had full access to all of the data in the study and can take responsibility for the integrity of the data and the accuracy of the data analysis.

Transparency declaration

The lead author affirms that this manuscript is an honest, accurate, and transparent account of the study being reported; that no important aspects of the study have been omitted; and that any discrepancies from the study as planned have been explained.

Funding

The model was based on work previously funded by Aquarius Population Health[18,19]

KT, EA, KT and HC did not receive any funding from O'Neill review to undertake this project. HC was funded by the National Institute for Health Research Health Protection Research Unit (NIHR HPRU) in Evaluation of Interventions at the University of Bristol in partnership with Public Health England (PHE). The views expressed are those of the author(s) and not necessarily those of the NHS, the NIHR, the Department of Health or Public Health England.

Competing interests

KT reports grants from EPSRC, during the conduct of the study; personal fees from Aquarius Population Health, other from WHO, grants from Guys and St Thomas Charity , outside the submitted work; and I am an editor of Sexually Transmitted Infections .; HC reports grants from NIHR, during the conduct of the study; other from Sanofi Pasteur, outside the submitted work; EA reports no compensation for the submitted work, and grants from Cepheid, Atlas Genetics, St Georges University of London, Enigma Diagnostics, and AstraZeneca, outside the submitted work; AM reports personal fees from Department of Health, non-financial support from Wellcome Trust, outside the submitted work; NW reports PHE's AMRHAI Reference Unit receiving financial support from Achaogen Inc., Allecra Antiinfectives GmbH, Amplex, AstraZeneca UK Ltd, Becton Dickinson Diagnostics, BSAC,

For peer review only - http://bmjopen.bmj.com/site/about/guidelines.xhtml

Cepheid, Check-Points B.V., Cubist Pharmaceuticals, Department of Health, Enigma Diagnostics, Food Standards Agency, GlaxoSmithKline Services Ltd, Henry Stewart Talks, IHMA Ltd, Merck Sharpe & Dohme Corp., Meiji Seika Kiasya Ltd, Momentum Biosciences Ltd, Nordic Pharma Ltd, Norgine Pharmaceuticals, Rempex Pharmaceuticals Ltd, Rokitan Ltd, Smith & Nephew UK Ltd, Trius Therapeutics, VenatoRx and Wockhardt Ltd, outside the submitted work. DM and HF: no conflicts to declare.

Ethical approval

Ethical approval was not required for this research, which uses routinely collected data and data from other studies.

Data sharing

Details of the model data inputs and other assumptions are provided in the methods and supporting parameters table. The model is available from http://amr-review.org/file/429 and researchers interested in further details may contact the corresponding author at katy.turner@bristol.ac.uk

Copyright

The Corresponding Author has the right to grant on behalf of all authors and does grant on behalf of all authors, an exclusive licence on a worldwide basis to the BMJ Publishing Group Ltd to permit this article (if accepted) to be published in BMJ editions and any other BMJPGL products and sublicences such use and exploit all subsidiary rights, as set out in our licence.

For peer review only

Figure 1: Number of gonorrhoea diagnoses reported in England, 2006 – 2015, with the change in recommended first line antibiotic treatment shown.

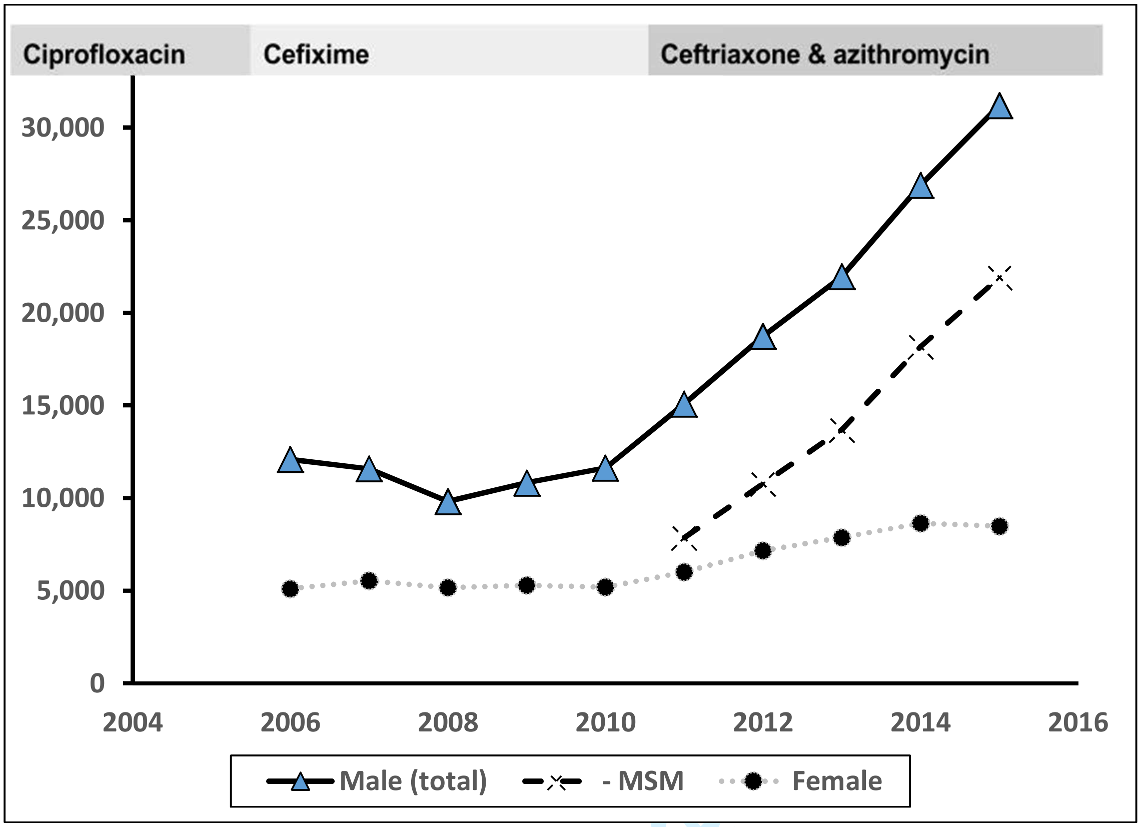

Legend: Data from Public Health England, Annual STI Data Tables https://www.gov.uk/government/statistics/sexually-transmitted-infections-stis-annual-data-tables

For peer review only - http://bmjopen.bmj.com/site/about/guidelines.xhtml

Figure 2 Current patient pathways for gonorrhoea

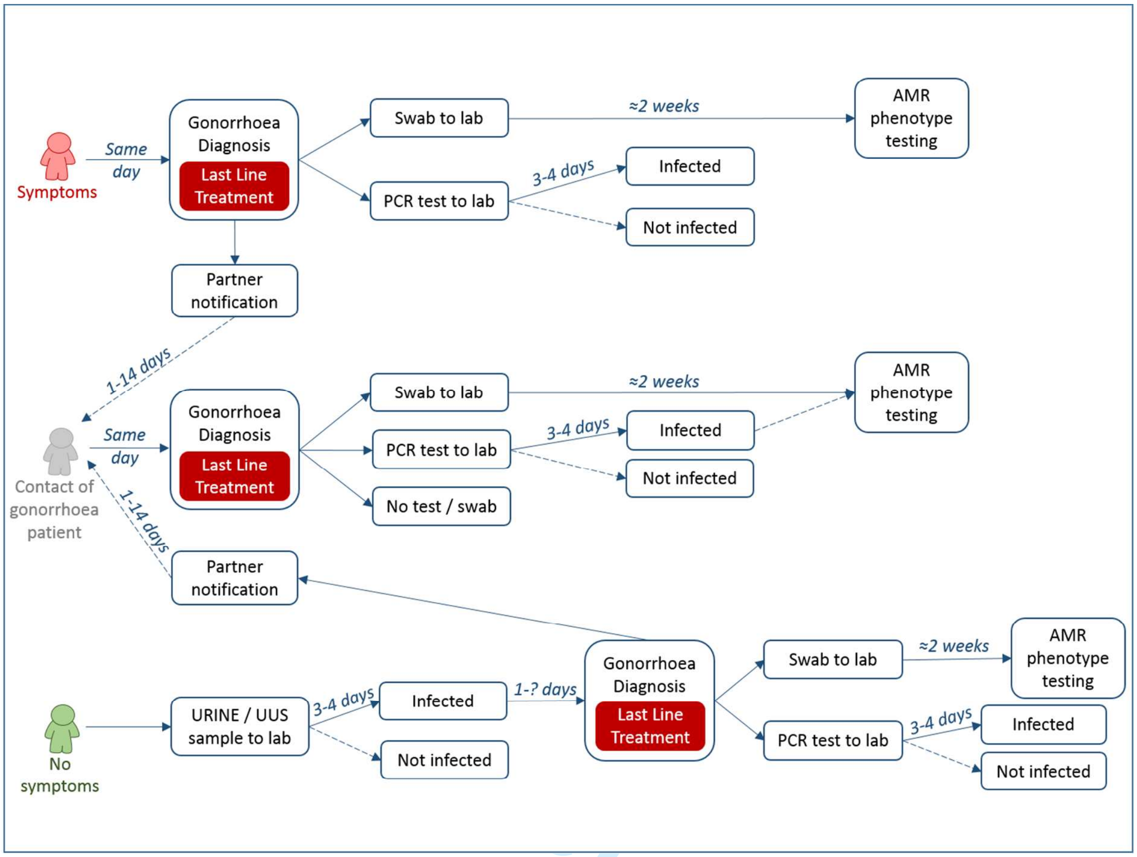

For peer review only - http://bmjopen.bmj.com/site/about/guidelines.xhtml

Figure 3 Patient pathway diagram to illustrate the flow for heterosexual males under A) current care, B) antimicrobial resistance point-of-care test

Legend: In scenario A, all diagnosed cases are treated with ceftriaxone plus azithromycin. In scenario B, diagnosed cases are treated according to resistance profile: AMR cases with ceftriaxone plus azithromycin; non-AMR with ciprofloxacin. Numbers of AMR and non-AMR infection are based on current levels of ciprofloxacin resistance observed in GRASP surveillance data, 2014. Illustrated based on 100,000 heterosexual men attending a genitourinary medicine clinic.

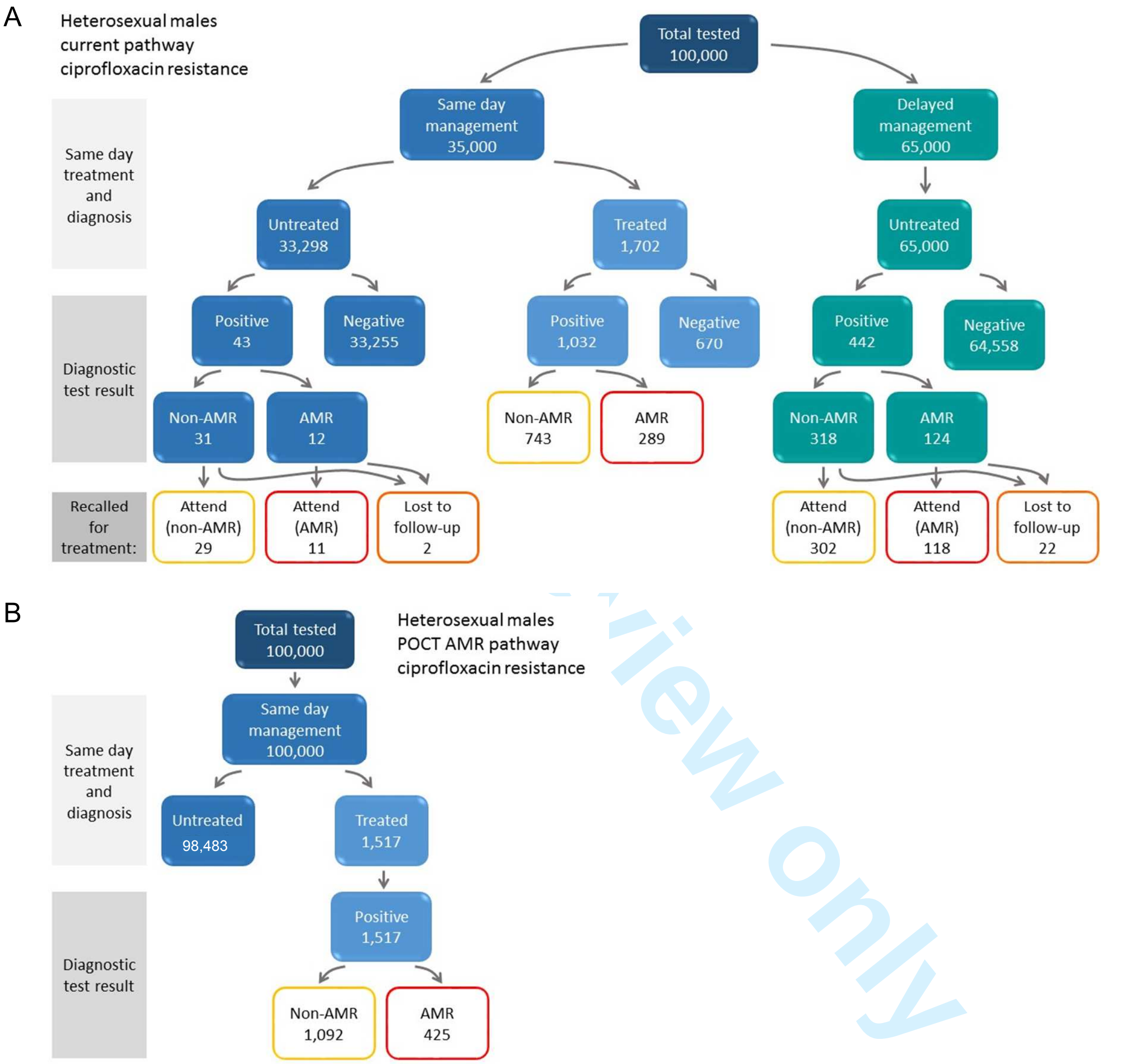

For peer review only

For peer review only - http://bmjopen.bmj.com/site/about/guidelines.xhtml

Table 1 Principal results comparing use of an antimicrobial resistance point-of-care test (AMR POCT) for ciprofloxacin (Scenario 3a) or penicillin resistance (Scenario 3b) against current testing practice (standard laboratory testing, no POCT) for the management of gonorrhoea (Scenario 1), assuming the current attendance at GUM clinic annually

	Heterosexual male	MSM	Female	Overall
Considering use of POCT test for ciprofloxacin resistance				
Annual ceftriaxone treatments				
Current (scenario 1)	7690	17691	8050	33431
AMR POCT (scenario 3a)	2188	7933	1257	11378
Reduction under scenario 3a	5502	9759	6793	22054
Percentage reduction in ceftriaxone	72%	55%	84%	66%
Proportion treated same day				
Current (scenario 1)	68%	63%	21%	54%
AMR POCT (scenario 3a)	100%	100%	100%	100%
Increase under scenario 3a	32%	37%	79%	46%
Mean time to treatment (days)				
Current (scenario 1)	1.5	1.8	3.9	2.2
AMR POCT (scenario 3a)	0.0	0.0	0.0	0.0
Reduction under scenario 3a	1.5	1.8	3.9	2.2
Persons lost to follow up (untreated)				
Current (scenario 1)	125	338	329	792
AMR POCT (scenario 3a)	0	0	0	0
Considering use of POCT test for penicillin resistance				
Annual ceftriaxone treatments*				
Current (scenario 1)	7690	17691	8050	33431
AMR POCT (scenario 3b)	1407	4688	838	6932
Reduction under scenario 3b	6283	13004	7212	26499
Percentage reduction in ceftriaxone	82%	74%	90%	79%

*All other outcomes same as for use of POCT for ciprofloxacin resistance. MSM, men who have sex with men. Results for strategy 2 not shown – equivalent to strategy 3 except for choice of antibiotic treatment. Results for 3b also equivalent to 3a for outcomes except reduction in ceftriaxone treatments.

Definitions

GUM: Genitourinary medicine clinic, POCT: Point of care test, AMR: Antimicrobial resistance, MSM: Men who have sex with men

For peer review only - http://bmjopen.bmj.com/site/about/guidelines.xhtml

Table 2 Cost of testing and treatment* when using an antimicrobial resistance point-of-care test (AMR POCT) for ciprofloxacin resistance (strategy 3a) compared with current practice

	Heterosexual male	MSM	Female	Overall
Annual cost of testing				
Current	£69,784,517	£20,358,694	£105,826,467	£195,969,677
AMR POCT	£82,415,040	£23,338,080	£124,653,600	£230,406,720
Increased cost with AMR POCT	£12,630,523	£2,979,386	£18,827,133	£34,437,043

*The model assumes that the additional cost of AMR POCT (£25) is simply added to the cost of attendance, and is not offset by reductions in the number of gonorrhoea infections, by reduced treatment costs (as some patients are treated with cheaper antibiotics), or by reduced use of other tests (such as microscopy or culture of all swabs).

For peer review only

For peer review only - http://bmjopen.bmj.com/site/about/guidelines.xhtml

REFERENCE LIST

1. WHO. Antimicrobial resistance global report on surveillance: 2014 summary. . 2014.
2. Health UGDo. UK Five Year Antimicrobial Resistance Strategy 2013 to 2018., 2011.
3. Public Health England. STI diagnoses & rates by gender, sexual risk & age group, 2011 - 2015, 2016.
4. Public Health England. STI diagnoses & rates by gender, sexual risk & age group, 2010 - 2014, 2015.
5. Korenromp EL, Sudaryo MK, de Vlas SJ, et al. What proportion of episodes of gonorrhoea and chlamydia becomes symptomatic? *Int J STD AIDS* 2002; **13**(2): 91-101.
6. Yeh JM, Hook EW, 3rd, Goldie SJ. A refined estimate of the average lifetime cost of pelvic inflammatory disease. *Sex Transm Dis* 2003; **30**(5): 369-78.
7. Chesson HW, Pinkerton SD. Sexually transmitted diseases and the increased risk for HIV transmission: implications for cost-effectiveness analyses of sexually transmitted disease prevention interventions. *J Acquir Immune Defic Syndr* 2000; **24**(1): 48-56.
8. Bignell C, Fitzgerald M, Guideline Development G, British Association for Sexual H, Hiv UK. UK national guideline for the management of gonorrhoea in adults, 2011. *Int J STD AIDS* 2011; **22**(10): 541-7.
9. England. PH. Surveillance of antimicrobial resistance in *Neisseria gonorrhoeae*. Key findings from the 'Gonococcal resistance to antimicrobials surveillance programme' (GRASP) and related surveillance data, 2014.
10. Public Health England GRASP Steering Committee and BASHH CEG. Gonorrhoea Treatment Position Statement 2015.
11. England PH. GRASP Report 2016, 2016.
12. Hemarajata P, Yang S, Soge OO, Humphries RM, Klausner JD. Performance and Verification of a Real-Time PCR Assay Targeting the gyrA Gene for Prediction of Ciprofloxacin Resistance in Neisseria gonorrhoeae. *J Clin Microbiol* 2016; **54**(3): 805-8.
13. Chaudhry U, Ray K, Bala M, Saluja D. Mutation patterns in gyrA and parC genes of ciprofloxacin resistant isolates of Neisseria gonorrhoeae from India. *Sex Transm Infect* 2002; **78**(6): 440-4.
14. De Silva D, Peters J, Cole K, et al. Whole-genome sequencing to determine transmission of Neisseria gonorrhoeae: an observational study. *Lancet Infect Dis* 2016; **16**(11): 1295-303.
15. Allen VG, Melano RG. Whole-genome sequencing-new tools for gonorrhoea control. *Lancet Infect Dis* 2016; **16**(11): 1214-5.
16. Aanensen DM, Feil EJ, Holden MT, et al. Whole-Genome Sequencing for Routine Pathogen Surveillance in Public Health: a Population Snapshot of Invasive Staphylococcus aureus in Europe. *MBio* 2016; **7**(3).
17. Vickerman P, Watts C, Alary M, Mabey D, Peeling RW. Sensitivity requirements for the point of care diagnosis of Chlamydia trachomatis and Neisseria gonorrhoeae in women. *Sexually Transmitted Infections* 2003; **79**(5): 363-7.
18. Turner KM, Round J, Horner P, et al. An early evaluation of clinical and economic costs and benefits of implementing point of care NAAT tests for *Chlamydia trachomatis* and *Neisseria gonorrhoea* in genitourinary medicine clinics in England. *Sex Transm Infect* 2014; **90**(2): 104-11.
19. Adams EJ, Ehrlich A, Turner KM, et al. Mapping patient pathways and estimating resource use for point of care versus standard testing and treatment of chlamydia and gonorrhoea in genitourinary medicine clinics in the UK. *BMJ Open* 2014; **4**(7): e005322.
20. Mercer CH, Macdonald N, Shirley MDF, et al. The Maximising STI Control (MSTIC) webtool: a new approach to facilitate the planning of services for sexually transmitted infections to maximise public health benefit. *Lancet* 2013; **382**: 6-.
21. Kent CK, Chaw JK, Wong W, et al. Prevalence of rectal, urethral, and pharyngeal chlamydia and gonorrhea detected in 2 clinical settings among men who have sex with men: San Francisco, California, 2003. *Clin Infect Dis* 2005; **41**(1): 67-74.
22. Monitor and NHS England. 2014/15 National Tariff Payment System, 2013.

For peer review only - http://bmjopen.bmj.com/site/about/guidelines.xhtml

23. Grad YH, Goldstein E, Lipsitch M, White PJ. Improving Control of Antibiotic-Resistant Gonorrhea by Integrating Research Agendas Across Disciplines: Key Questions Arising From Mathematical Modeling. *J Infect Dis* 2016; **213**(6): 883-90.
24. Garnett GP. The theoretical impact and cost-effectiveness of vaccines that protect against sexually transmitted infections and disease. *Vaccine* 2014; **32**(14): 1536-42.
25. Regnier SA, Huels J. Potential impact of vaccination against Neisseria meningitidis on Neisseria gonorrhoeae in the United States: results from a decision-analysis model. *Hum Vaccin Immunother* 2014; **10**(12): 3737-45.
26. Whelan J, Klovstad H, Haugen IL, Holle MR, Storsaeter J. Ecologic Study of Meningococcal B Vaccine and Neisseria gonorrhoeae Infection, Norway. *Emerg Infect Dis* 2016; **22**(6): 1137-9.
27. O'Neill J. Tackling Drug-Resistant Infections Globally: final report and recommendations. The review on antimicrobial resistance, 2016.
28. White PJ, Ward H, Cassell JA, Mercer CH, Garnett GP. Vicious and virtuous circles in the dynamics of infectious disease and the provision of health care: gonorrhea in Britain as an example. *J Infect Dis* 2005; **192**(5): 824-36.
29. McAdams D. Resistance diagnosis and the changing epidemiology of antibiotic resistance. *Ann N Y Acad Sci* 2017; **1388**(1): 5-17.
30. Harding-Esch EM, Hegazi A, Okolo O, et al. P2.163 Do “In-Clinic” Molecular and Non-Molecular Rapid Tests Improve Patient Management? *Sexually Transmitted Infections* 2013; **89**(Suppl 1): A137-A8.

For peer review only

For peer review only - http://bmjopen.bmj.com/site/about/guidelines.xhtml

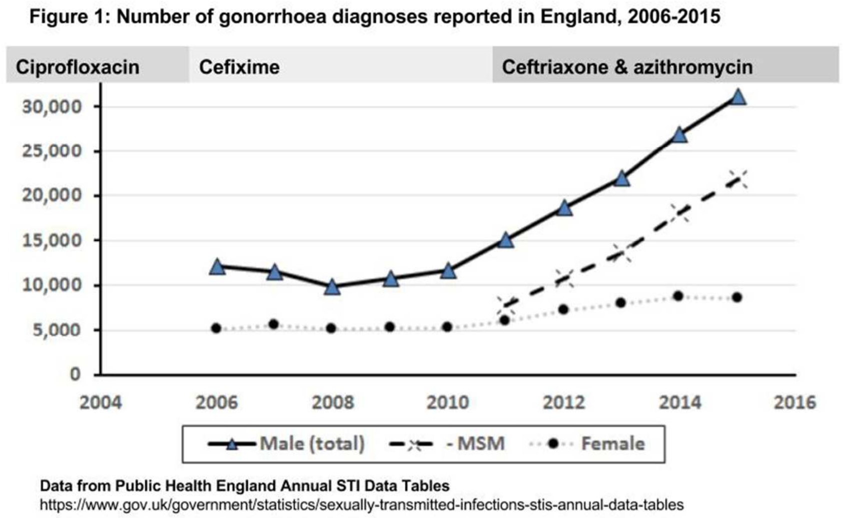

338x190mm (72 x 72 DPI)

review only

Supplementary information for:

Analysis of the potential for point-of-care test to enable individualised treatment of infections caused by antimicrobial-resistant and susceptible strains of *Neisseria gonorrhoeae*

Katy ME Turner, Hannah Christensen, Elisabeth J Adams, David McAdams, Helen Fifer, Anthony McDonnell, Neil Woodford

Table A 1 Current prevalence of antimicrobial resistance to potential treatments for gonorrhoea

Drug	Class	Prevalence of resistance in GRASP 2014 isolates[1]			
		Heterosexual men	MSM	Women	Overall
Ceftriaxone	Cephalosporin (3rd generation)	0	0	0	0
Penicillin	β-lactam	18%	26%	10%	23%
Ciprofloxacin	Fluoroquinolone	28%	44%	15%	37%
Azithromycin	Macrolide	0.0%	1.4%	0.5%	1.0%

Current first line (and last-line) therapy is intramuscular ceftriaxone with azithromycin 1g orally. MSM, men who have sex with men.
GRASP: Gonococcal Resistance to Antimicrobial Surveillance Programme

For peer review only - http://bmjopen.bmj.com/site/about/guidelines.xhtml

Table A2 Model parameters

Baseline model parameters	Current			AMR POCT		
	Heterosexual men	MSM	Women	Heterosexual men	MSM	Women
Initial population size[2]	515,094	145,863	779,085	515,094	145,863	779,085
Proportion entering same day management pathway	35%	33%	48%	100%	100%	100%
Proportion infected with gonorrhoea (of total tested)[2]	1.5%	12.4%	1.1%	1.5%	12.4%	1.1%
Proportion of those in same day pathway infected with gonorrhoea	3.1%	26.0%	1.0%	1.5%	12.4%	1.1%
Proportion of delayed management infected with gonorrhoea	0.7%	5.6%	1.2%	-	-	-
Relative risk infection gonorrhoea in same day vs delayed pathway	4.52	4.63	0.82	-	-	-
Proportion in same day pathway who are infected & treated on same day	96%	90%	50%	100%	100%	100%
Proportion of same day pathway treated presumptively for gonorrhoea	5.0%	25.0%	2.0%	1.5%	12.4%	1.1%
Proportion who attend for treatment after lab test result (of those who wait for lab test results, i.e. asymptomatic group (Figure 3))	95%	95%	95%	100%	100%	100%
Proportion treated with last line therapy[3]	100%	100%	100%	28%	44%	15%
Cost of first attendance[4 5]	£135	£135	£135	£135	£135	£135
Cost of follow-up attendance[4 5]	£104	£104	£104	£104	£104	£104
Additional cost of AMR POCT (assumed)[6]	£25	£25	£25	£25	£25	£25

AMR POCT, antimicrobial resistance point of care test; MSM, men who have sex with men.

For peer review only

For peer review only - http://bmjopen.bmj.com/site/about/guidelines.xhtml

References

1 Adams EJ, Ehrlich A, Turner KM, Shah K, Macleod J, Goldenberg S, et al. Mapping patient pathways and estimating resource use for point of care versus standard testing and treatment of chlamydia and gonorrhoea in genitourinary medicine clinics in the UK. BMJ Open 2014; 4(7): e005322.

2 Public Health England. STI diagnoses & rates by gender, sexual risk & age group, 2010 - 2014, 2015.

3 England. PH. Surveillance of antimicrobial resistance in *Neisseria gonorrhoeae*. Key findings from the 'Gonococcal resistance to antimicrobials surveillance programme' (GRASP) and related surveillance data, 2014.

4 Monitor and NHS England. 2014/15 National Tariff Payment System, 2013.

5 Health PHEatDo. HIV, sexual and reproductive health: current issues bulletin2013. Available from: https://www.gov.uk/government/uploads/system/uploads/attachment_data/file/259087/HIV_Sexual_and_Reproductive_Health_bulletin-issue1nov2013.pdf.

6 Turner KM, Round J, Horner P, Macleod J, Goldenberg S, Deol A, et al. An early evaluation of clinical and economic costs and benefits of implementing point of care NAAT tests for *Chlamydia trachomatis* and *Neisseria gonorrhoea* in genitourinary medicine clinics in England. Sex Transm Infect 2014; 90(2): 104-11.

For peer review only

Scenario

	Day
Initial test	0
Clinical exam or not	0
Same day treatment & diagnosis	0
Infection status	
	0
Proportion treated with last line therapy	0
Swab sent for resistance typing (1)	0
Lab results	2
Recalled for treatment	2
Attend for treatment	5
Swab sent for resistance typing (2)	

	Symp.	Asympt
Infected	5538	2277
Resistant	997	410

Infec
22
182
Alternative regimen

Posi
182

182

Atte
173
173

For peer review only - http://bmjopen.bmj.com/site/about/guidelines.xhtml

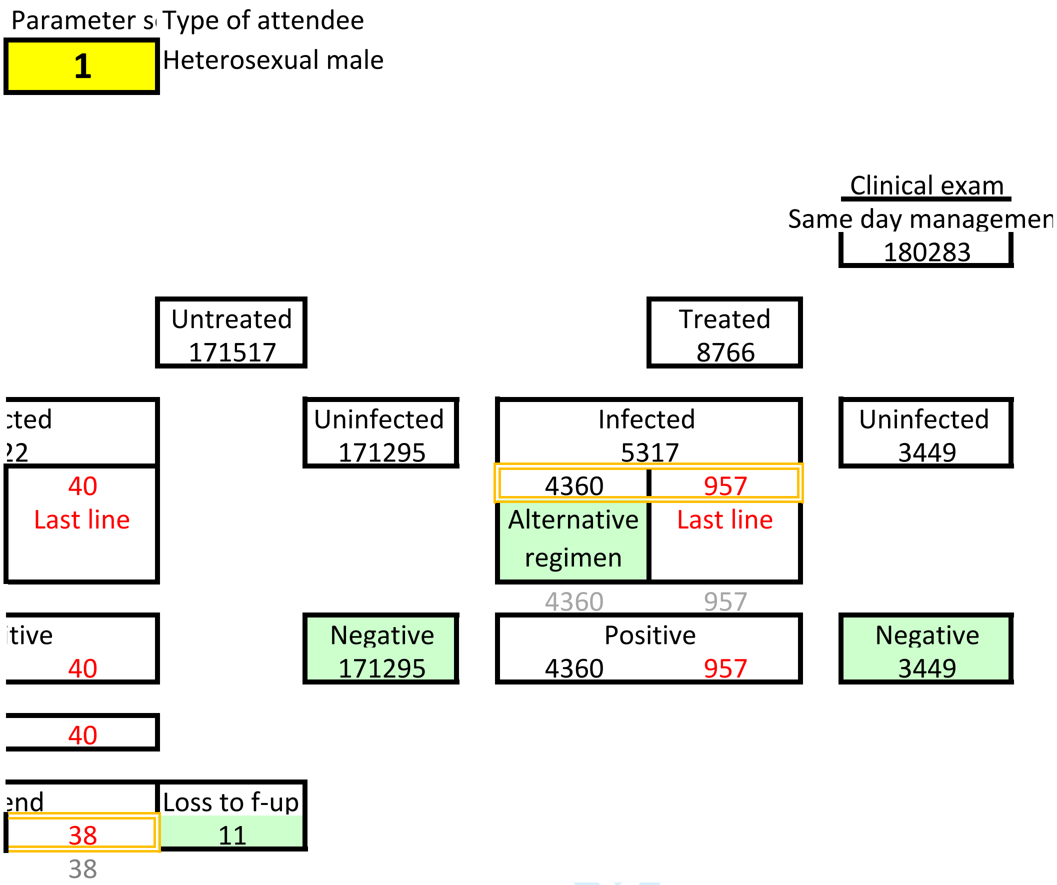
Parameter s
Type of attendee
1
Heterosexual male
Clinical exam
Same day managemen
180283
Untreated
171517
Treated
8766
cted
22
Uninfected
171295
Infected
5317
Uninfected
3449
40
Last line
4360
957
Alternative regimen
Last line
4360
957
tive
40
Negative
171295
Positive
4360
957
Negative
3449
40
end
Loss to f-up
38
11
38

view only

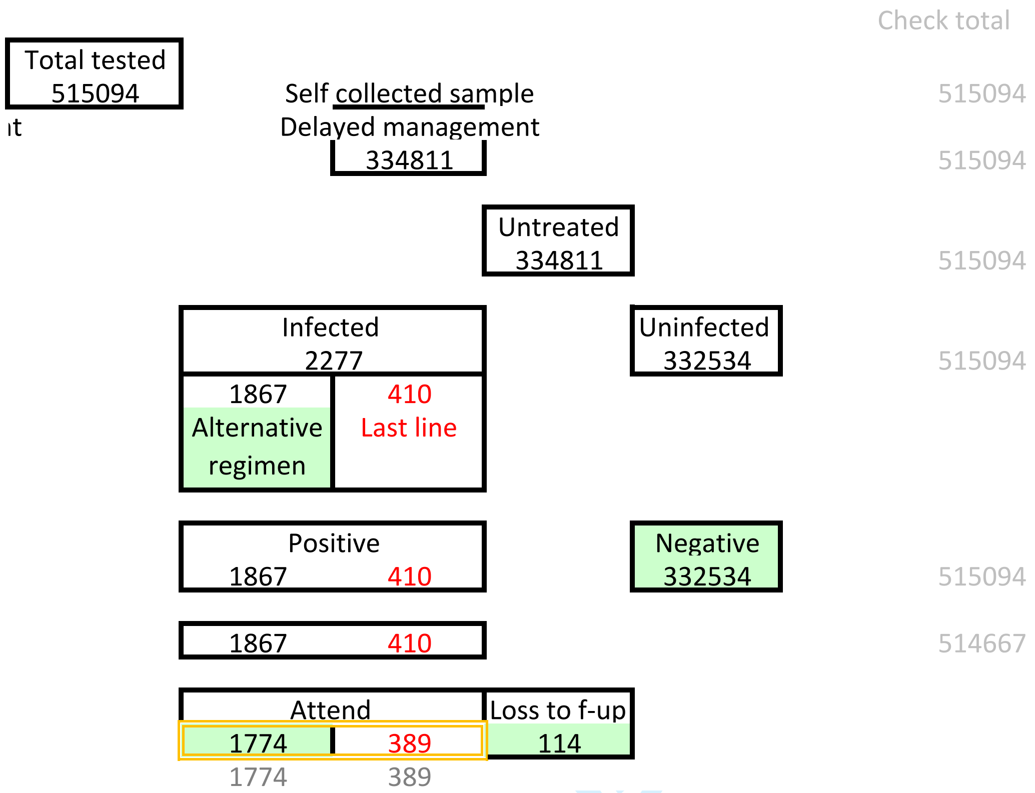

could we treat with cipro rather than

view only

Total number follow-up attendances	2373
Number infected	7815
Total visits (for positives)	10188
Total days waited for appropriate treatment	11867
Number lost to follow up (untreated)	125
Number of people who are given ceftriaxone, current and POCT scenario	7690
Number of people who are given ceftriaxone, POCT AMR scenario	1384
Number of people who are given ceftriaxone, given parameter set	7690
% treated on same day	12%
Number treated on same day	5317
Mean number of days to treatment	1.5

For peer review only

For peer review only

For peer review only

		Live values
Do not edit live cells!		1
Total sexual health screens	2	36%
Total New STI diagnoses	3	39%
Proportion symptomatic	4	35%
Proportion of those symptomatic infected with gonorrhea	7	3%
Proportion of asymptomatic infected with gonorrhoea	8	1%
Relative risk infection gonorrhoea if symptomatic cf asymptomatic	9	4.52
Overall proportion infected GC (asympt +sympt inf/attend)	10	2%
Proportion of symptomatic & infected who are treated on same day as test	19	96%
Proportion of symptomatics treated presumptively for gonorrhoea	21	5%
Proportion of those positive who attend for treatment after lab test result	26	95%
Proportion of those diagnosed & infected who attend for test of cure	27	50%
Initial population size	28	515,094
Proportion treated with last line therapy	29	18%
Proportion reattend for retreatment after resistance diagnosed from	31	95%
Cost of first attendance	32	£ 135
Cost of follow-up attendance	33	£ 104
Type of test used (1 - standard care, 2- POCT, 3 POCT AMR)	34	1
Ratio of test cost 3 (AMR POCT) to 2 (POCT)	35	2
Cost of test 2 (POCT) (additional)	36	25

For peer review only

Baseline model parameters SC te			POCT test baseline			POCT AMR baseline	
Heterosexual male	MSM	Female	Heterosexual male	MSM	Female	Heterosexual male	MSM
**1**	**2**	**3**	**4**	**5**	**6**	**7**	**8**
36.4%	10.3%	53.2%	36.4%	10.3%	53.2%	36.4%	10.3%
38.6%	13.0%	48.4%	38.6%	13.0%	48.4%	38.6%	13.0%
**35.0%**	**33.0%**	**48.0%**	**100.0%**	**100.0%**	**100.0%**	**100.0%**	**100.0%**
3.1%	26.0%	1.0%	1.5%	12.4%	1.1%	1.5%	12.4%
0.7%	5.6%	1.2%	1.5%	12.4%	1.1%	1.5%	12.4%
**4.52**	**4.63**	**0.82**	**4.52**	**4.63**	**0.82**	**4.52**	**4.63**
1.5%	12.4%	1.1%	1.5%	12.4%	1.1%	1.5%	12.4%
96%	90%	50%	100%	100%	100%	100%	100%
5%	25%	2%	1.5%	12.4%	1.1%	1.5%	12.4%
95%	95%	95%	100%	100%	100%	100%	100%
50%	50%	50%	100%	100%	100%	100%	100%
**515,094**	**145,863**	**779,085**	**515,094**	**145,863**	**779,085**	**515,094**	**145,863**
18%	26%	10%	18%	26%	10%	18%	26%
95%	95%	95%	95%	95%	95%	0%	0%
135	135	135	135	135	135	135	135
104	104	104	104	104	104	104	104
1	1	1	2	2	2	3	3
2	2	2	2	2	2	2	2
25	25	25	25	25	25	25	25

	Simplified model parameters (live calcula			Baseline model param	
Female	Heterosexual male	MSM	Female	Heterosexual male	MSM
**9**	**90**	**91**	**92**	**100**	**200**
53.2%	36.4%	10.3%	53.2%	36.4%	10.3%
48.4%	38.6%	13.0%	48.4%	38.6%	13.0%
**100.0%**	**35.0%**	**33.0%**	**48.0%**	**35.0%**	**33.0%**
1.1%	3.1%	26.0%	1.0%	3.1%	26.0%
1.1%	0.7%	5.6%	1.2%	0.7%	5.6%
**0.82**	**4.52**	**4.63**	**0.82**	**4.5**	**4.6**
1.1%	1.5%	12.4%	1.1%	1.5%	12.4%
100%	96%	90%	50%	96%	90%
1.1%	5%	25%	2%	5%	25%
100%	100%	100%	100%	100%	100%
100%	100%	100%	100%	100%	100%
**779,085**	**515,094**	**145,863**	**779,085**	**515,094**	**145,863**
10%	0%	0%	0%	5%	30%
0%	95%	95%	95%	95%	95%
135	135	135	135	**160**	**160**
104	104	104	104	**120**	**120**
3	1	1	1	1	1
2	2	2	2	2	2
25	25	25	25	25	25

ıeters (live	England 2014 numbers			Source
Female	Heterosexu al male	MSM	Female	
**300**				
53.2%	**515,094**	**145,863**	**779,085**	**PHE 2014**
48.4%	**143,244**	**48,206**	**210,843**	**PHE 2014**
**48.0%**	180,283	48,135	373,961	
1.0%	5,538	12,529	3,600	
1.2%	2,277	5,500	4,779	
**0.82**				
1.1%	**7,815**	**18,029**	**8,379**	**GRASP**
50%	5,317	11,276	1,800	
2%	8,766	11,878	9,112	
100%	guess from clinics			
100%				
**779,085**				
5%				
95%				
160	check PBR tariff costs			
120	check PBR tariff costs			
1				
2				
25				

For peer review only

Weblink
https://www.gov.uk/government/uploads/syst
https://www.gov.uk/government/uploads/syst
https://www.gov.uk/government/uploads/syst

review only

Comments	Other info
Total sexual health screens 2014 (including gonorrhoea tests)	
New episodes of STI 2014	
MSTIC data based on Cath Mercer study (as reported in	533 Men, 731 Women, 98 MSM

em/uploads/attachment_data/file/476582/GRASP_2014_report_final_111115.pdf

% of true symptomatic infections are correctly treated presumptively on basis of symptoms/mi

w only

For peer review only

Public Health England

Table 4 (a): Number of all STI diagnoses & services in Englar

Data type: service data

**New STI diagnoses**		
Chancroid / LGV / Donovanosis	C1, C2, C3	**Male** (total)
		- Heterosexual
		- MSM
		**Female** (total)
		- Heterosexual
		- WSW
		**Total**
Chlamydia - total ¹	-	**Male** (total)
		- Heterosexual
		- MSM
		**Female** (total)
		- Heterosexual
		- WSW
		**Total**
¹ Chlamydia - GUM services	C4°, C4Aˇ, C4Bˇ, C4Cˇ	**Male** (total)
		- Heterosexual
		- MSM
		**Female** (total)
		- Heterosexual
		- WSW
		**Total**
¹ Chlamydia - community services	-	**Male** (total)
		- Heterosexual
		- MSM
		**Female** (total)
		- Heterosexual
		- WSW
		**Total**
Gonorrhoea	B°, B1ˇ, B2ˇ, B5ˇ	**Male** (total)
		- Heterosexual
		- MSM
		**Female** (total)
		- Heterosexual
		- WSW
		**Total**
Herpes: anogenital herpes (1st episode)	C10A	**Male** (total)
		- Heterosexual
		- MSM
		**Female** (total)
		- Heterosexual
		- WSW
		**Total**
HIV: new diagnosis - total ² **	H1°, H1A°, H1B°, E1Aˇ, E2Aˇ, E3A1ˇ	**Male** (total)
		- Heterosexual
		- MSM
		**Female** (total)
		- Heterosexual
		- WSW

		**Total**
² HIV: new diagnosis	H1A°	**Male** (total)
- acute infections **		- Heterosexual
		- MSM
		**Female** (total)
		- Heterosexual
		- WSW
		**Total**
² HIV: new diagnosis	H1B°, E3A1˘	**Male** (total)
- AIDS defined **		- Heterosexual
		- MSM
		**Female** (total)
		- Heterosexual
		- WSW
		**Total**
Molluscum contagiosum **	C12	**Male** (total)
		- Heterosexual
		- MSM
		**Female** (total)
		- Heterosexual
		- WSW
		**Total**
Non-specific genital infection (NSGI)	C4N°, C4H˘	**Male** (total)
		- Heterosexual
		- MSM
		**Female** (total)
		- Heterosexual
		- WSW
		**Total**
**New STI diagnoses (continued)**		
Pelvic inflammatory disease (PID) & epididymitis: non-specific **	C5A°, C5˘	**Male** (total)
		- Heterosexual
		- MSM
		**Female** (total)
		- Heterosexual
		- WSW
		**Total**
Chlamydial PID & epididymitis (included in chlamydia total)	C5A°+C4°, C4B˘	**Male** (total)
		- Heterosexual
		- MSM
		**Female** (total)
		- Heterosexual
		- WSW
		**Total**
Gonococcal PID & epididymitis (included in gonorrhoea total)	C5A°+B°, B5	**Male** (total)
		- Heterosexual
		- MSM
		**Female** (total)
		- Heterosexual
		- WSW
		**Total**
Scabies / pediculosis pubis **	C8, C9	**Male** (total)
		- Heterosexual
		- MSM
		**Female** (total)
		- Heterosexual
		- WSW
		**Total**

For peer review only

Syphilis: primary, secondary, & early latent	A1, A2, A3	**Male** (total)
		- Heterosexual
		- MSM
		**Female** (total)
		- Heterosexual
		- WSW
		**Total**
Trichomoniasis	C6A	**Male** (total)
		- Heterosexual
		- MSM
		**Female** (total)
		- Heterosexual
		- WSW
		**Total**
Warts: anogenital warts (1st episode)	C11A	**Male** (total)
		- Heterosexual
		- MSM
		**Female** (total)
		- Heterosexual
		- WSW
		**Total**
**Other STI diagnoses**		
Epidemiological treatment of suspected chlamydia	C4Eˇ	**Male** (total)
		- Heterosexual
		- MSM
		**Female** (total)
		- Heterosexual
		- WSW
		**Total**
Epidemiological treatment of suspected gonorrhoea	B4ˇ	**Male** (total)
		- Heterosexual
		- MSM
		**Female** (total)
		- Heterosexual
		- WSW
		**Total**
Epidemiological treatment of NSGI	C4Iˇ	**Male** (total)
		- Heterosexual
		- MSM
		**Female** (total)
		- Heterosexual
		- WSW
		**Total**
Epidemiological treatment of suspected syphilis	A9ˇ	**Male** (total)
		- Heterosexual
		- MSM
		**Female** (total)
		- Heterosexual
		- WSW
		**Total**
Herpes: anogenital herpes (recurrent episode)	C10B	**Male** (total)
		- Heterosexual
		- MSM
		**Female** (total)
		- Heterosexual
		- WSW
		**Total**
HIV: subsequent presentation	H2°, E1Bˇ, E2Bˇ,	**Male** (total)

For peer review only

	E3A2ˇ, E3Bˇ	- Heterosexual
		- MSM
		**Female** (total)
		- Heterosexual
		- WSW
		**Total**
Ophthalmia neonatorum	C5B°, B3ˇ, C4Dˇ	**Male** (total)
		- Heterosexual
		- MSM
		**Female** (total)
		- Heterosexual
		- WSW
		**Total**
**Other STI diagnoses (continued)**		
Syphilis: congenital syphilis aged under 2	A7A°, A7ˇ	**Male** (total)
		- Heterosexual
		- MSM
		**Female** (total)
		- Heterosexual
		- WSW
		**Total**
Syphilis: congenital syphilis aged 2 or over	A7A°, A8ˇ	**Male** (total)
		- Heterosexual
		- MSM
		**Female** (total)
		- Heterosexual
		- WSW
		**Total**
Syphilis: late	A4, A5, A6	**Male** (total)
		- Heterosexual
		- MSM
		**Female** (total)
		- Heterosexual
		- WSW
		**Total**
Warts: anogenital warts (recurrent episode)	C11D°, C11Bˇ, C11Cˇ	**Male** (total)
		- Heterosexual
		- MSM
		**Female** (total)
		- Heterosexual
		- WSW
		**Total**
**Other GUM services diagnoses**		
Candidosis: anogenital	C7°, C7Aˇ	**Male** (total)
		- Heterosexual
		- MSM
		**Female** (total)
		- Heterosexual
		- WSW
		**Total**
Cervical cytology: minor abnormality	P4A	**Male** (total)
		- Heterosexual
		- MSM
		**Female** (total)
		- Heterosexual
		- WSW
		**Total**
Cervical cytology:	P4B	**Male** (total)

major abnormality		- Heterosexual
		- MSM
		**Female** (total)
		- Heterosexual
		- WSW
		**Total**
Epidemiological treatment of candidosis, vaginosis, vaginitis & balanitis	C7Bˇ	**Male** (total)
		- Heterosexual
		- MSM
		**Female** (total)
		- Heterosexual
		- WSW
		**Total**
Hepatitis A: acute infection	C15°	**Male** (total)
		- Heterosexual
		- MSM
		**Female** (total)
		- Heterosexual
		- WSW
		**Total**
Hepatitis B: first diagnosis	C13°, C13Aˇ	**Male** (total)
		- Heterosexual
		- MSM
		**Female** (total)
		- Heterosexual
		- WSW
		**Total**
Hepatitis C: first diagnosis	C14	**Male** (total)
		- Heterosexual
		- MSM
		**Female** (total)
		- Heterosexual
		- WSW
		**Total**
Other conditions requiring treatment at GUM	D2B	**Male** (total)
		- Heterosexual
		- MSM
		**Female** (total)
		- Heterosexual
		- WSW
		**Total**
Urinary tract infection	D2A	**Male** (total)
		- Heterosexual
		- MSM
		**Female** (total)
		- Heterosexual
		- WSW
		**Total**
Vaginosis / vaginitis / balanitis	C6B, C6C	**Male** (total)
		- Heterosexual
		- MSM
		**Female** (total)
		- Heterosexual
		- WSW
		**Total**
**Services provided**		
Cervical cytology performed	P4°	**Male** (total)
		- Heterosexual

For peer review only

		- MSM
		**Female** (total)
		- Heterosexual
		- WSW
		**Total**
Contraception (excluding condom provision)	P3	**Male** (total)
		- Heterosexual
		- MSM
		**Female** (total)
		- Heterosexual
		- WSW
		**Total**
Hepatitis B immune (not included in 'Total services provided')	P2I°	**Male** (total)
		- Heterosexual
		- MSM
		**Female** (total)
		- Heterosexual
		- WSW
		**Total**
Hepatitis B vaccination: 1st dose	P2A°, P2˘	**Male** (total)
		- Heterosexual
		- MSM
		**Female** (total)
		- Heterosexual
		- WSW
		**Total**
Hepatitis B vaccination: 2nd dose	P2B°	**Male** (total)
		- Heterosexual
		- MSM
		**Female** (total)
		- Heterosexual
		- WSW
		**Total**
Hepatitis B vaccination: 3rd dose	P2C°	**Male** (total)
		- Heterosexual
		- MSM
		**Female** (total)
		- Heterosexual
		- WSW
		**Total**
HPV vaccination: 1st dose	W1°	**Male** (total)
		- Heterosexual
		- MSM
		**Female** (total)
		- Heterosexual
		- WSW
		**Total**
HPV vaccination: 2nd dose	W2°	**Male** (total)
		- Heterosexual
		- MSM
		**Female** (total)
		- Heterosexual
		- WSW
		**Total**
HPV vaccination: 3rd dose	W3°	**Male** (total)
		- Heterosexual

For peer review only

		- MSM
		**Female** (total)
		- Heterosexual
		- WSW
		**Total**
Other episodes not requiring treatment	D3	**Male** (total)
		- Heterosexual
		- MSM
		**Female** (total)
		- Heterosexual
		- WSW
		**Total**
Partner notification: chlamydia	PNC°	**Male** (total)
		- Heterosexual
		- MSM
		**Female** (total)
		- Heterosexual
		- WSW
		**Total**
Partner notification: gonorrhoea	PNG°	**Male** (total)
		- Heterosexual
		- MSM
		**Female** (total)
		- Heterosexual
		- WSW
		**Total**
Partner notification: HIV	PNH°	**Male** (total)
		- Heterosexual
		- MSM
		**Female** (total)
		- Heterosexual
		- WSW
		**Total**
Partner notification: syphilis	PNS°	**Male** (total)
		- Heterosexual
		- MSM
		**Female** (total)
		- Heterosexual
		- WSW
		**Total**
**Services provided (continued)**		
Post exposure prophylaxis (sexual exposure)	PEPS°	**Male** (total)
		- Heterosexual
		- MSM
		**Female** (total)
		- Heterosexual
		- WSW
		**Total**
Testing: chlamydia tests (total) [3] (not included in 'Total services provided')	-	**Male** (total)
		- Heterosexual
		- MSM
		**Female** (total)
		- Heterosexual
		- WSW
		**Total**
[3] Chlamydia tests - GUM services (not included in 'Total services provided')	-	**Male** (total)
		- Heterosexual
		- MSM

For peer review only

Chlamydia tests from GUM services are not equal to the sum of source KC60/SHHAPT codes		**Female** (total)
		- Heterosexual
		- WSW
		**Total**
[3] Chlamydia tests - community services	-	**Male** (total)
		- Heterosexual
		- MSM
		**Female** (total)
		- Heterosexual
		- WSW
		**Total**
Testing: chlamydia test	T1°	**Male** (total)
		- Heterosexual
		- MSM
		**Female** (total)
		- Heterosexual
		- WSW
		**Total**
Testing: HIV tests (total)<br>(not included in 'Total services provided')<br><br>HIV tests total are not equal to the sum of source KC60/SHHAPT codes	-	**Male** (total)
		- Heterosexual
		- MSM
		**Female** (total)
		- Heterosexual
		- WSW
		**Total**
Testing: HIV test	P1A	**Male** (total)
		- Heterosexual
		- MSM
		**Female** (total)
		- Heterosexual
		- WSW
		**Total**
Testing: HIV test offered & refused	P1B	**Male** (total)
		- Heterosexual
		- MSM
		**Female** (total)
		- Heterosexual
		- WSW
		**Total**
Testing: HIV test not appropriate	P1C°	**Male** (total)
		- Heterosexual
		- MSM
		**Female** (total)
		- Heterosexual
		- WSW
		**Total**
**Services provided (continued)**		
Testing: sexual health screens [4]	S1˘, S2˘, T2°, T3°, T4°	**Male** (total)
		- Heterosexual
		- MSM
		**Female** (total)
		- Heterosexual
		- WSW
		**Total**
[4] without HIV test	S1˘	**Male** (total)
		- Heterosexual
		- MSM
		**Female** (total)

		- Heterosexual<br>- WSW<br>**Total**
[4] with HIV test	S2ˆˇ	**Male** (total)<br>- Heterosexual<br>- MSM<br>**Female** (total)<br>- Heterosexual<br>- WSW<br>**Total**
[4] chlamydia & gonorrhoea	T2°	**Male** (total)<br>- Heterosexual<br>- MSM<br>**Female** (total)<br>- Heterosexual<br>- WSW<br>**Total**
[4] chlamydia, gonorrhoea & syphilis	T3°	**Male** (total)<br>- Heterosexual<br>- MSM<br>**Female** (total)<br>- Heterosexual<br>- WSW<br>**Total**
[4] chlamydia, gonorrhoea, syphilis & HIV	T4°	**Male** (total)<br>- Heterosexual<br>- MSM<br>**Female** (total)<br>- Heterosexual<br>- WSW<br>**Total**
**Total new STI diagnoses**		**Male** (total)<br>- Heterosexual<br>- MSM<br>**Female** (total)<br>- Heterosexual<br>- WSW<br>**Total**
**Total other STI diagnoses**		**Male** (total)<br>- Heterosexual<br>- MSM<br>**Female** (total)<br>- Heterosexual<br>- WSW<br>**Total**
**Total other GUM services diagnoses**		**Male** (total)<br>- Heterosexual<br>- MSM<br>**Female** (total)<br>- Heterosexual<br>- WSW<br>**Total**
**Total services provided**		**Male** (total)<br>- Heterosexual

- MSM
**Female** (total)
- Heterosexual
- WSW
**Total**

Data for chlamydia, 'Total new STI diagnoses' & 'Total services provided' from 2012 onwards are not compa
(please see 'notes' section for further details).

Increases in numbers by sexual risk may be the result of improved data reporting (primarily affecting 2010-2011).

˘ KC60 code retired during 2011.

° SHHAPT code introduced during 2011.

** STI diagnoses (including HIV) not exclusively transmitted by sexual contact.

For peer review only

nd by gender & sexual risk, 2010 - 2014

Published 23/06/2015

2010	2011	2012	2013	2014
**419**	**385**	**354**	**476**	**674**
47	57	58	43	59
331	309	288	424	603
**74**	**96**	**60**	**77**	**78**
59	82	55	74	76
0	3	2	0	0
**493**	**481**	**414**	**553**	**753**
**76,985**	**77,427**	**85,986**	**85,434**	**85,106**
-	-	-	-	-
-	-	-	-	-
**111,924**	**109,875**	**119,548**	**120,719**	**120,008**
-	-	-	-	-
-	-	-	-	-
**189,400**	**187,664**	**207,797**	**207,851**	**206,774**
**48,018**	**52,049**	**51,542**	**53,630**	**55,807**
37,578	41,317	41,036	42,774	42,664
5,349	7,644	8,215	9,118	11,468
**46,080**	**50,048**	**45,870**	**48,642**	**51,045**
40,998	46,430	43,649	46,868	49,237
85	185	100	88	87
**94,152**	**102,117**	**97,425**	**102,289**	**106,865**
**28,967**	**25,378**	**34,444**	**31,804**	**29,299**
-	-	-	-	-
-	-	-	-	-
**65,844**	**59,827**	**73,678**	**72,077**	**68,963**
-	-	-	-	-
-	-	-	-	-
**95,248**	**85,547**	**110,372**	**105,562**	**99,909**
**11,634**	**15,081**	**18,583**	**21,751**	**26,575**
5,362	6,166	7,070	7,500	7,815
4,938	7,860	10,768	13,629	18,029
**5,198**	**6,007**	**6,992**	**7,664**	**8,379**
4,498	5,309	6,563	7,260	7,999
12	36	37	46	29
**16,843**	**21,090**	**25,576**	**29,419**	**34,958**
**11,582**	**11,926**	**12,094**	**12,277**	**11,889**
9,349	9,981	10,405	10,567	10,030
1,019	1,264	1,233	1,339	1,474
**18,101**	**19,226**	**19,770**	**20,069**	**19,883**
16,191	18,002	18,946	19,378	19,210
55	106	75	94	78
**29,698**	**31,154**	**31,864**	**32,349**	**31,777**
**3,031**	**3,330**	**3,088**	**3,112**	**3,247**
962	1,066	929	934	868
1,621	2,001	1,991	2,053	2,276
**1,314**	**1,232**	**1,042**	**958**	**907**
1,093	1,073	979	913	871
7	13	10	6	3

**4,347**	**4,562**	**4,131**	**4,071**	**4,155**
**-**	**242**	**240**	**226**	**219**
-	44	39	56	42
-	177	185	159	168
**-**	**57**	**53**	**54**	**36**
-	45	43	49	35
-	0	2	1	0
**-**	**299**	**293**	**280**	**255**
**171**	**270**	**220**	**194**	**204**
90	155	129	120	104
60	95	81	70	94
**86**	**132**	**106**	**91**	**85**
77	120	100	86	80
0	0	1	0	0
**257**	**402**	**326**	**285**	**289**
**8,825**	**8,594**	**8,267**	**7,852**	**7,473**
7,558	7,732	7,572	7,230	6,842
295	324	337	355	405
**3,783**	**3,618**	**3,617**	**3,425**	**3,141**
3,386	3,376	3,453	3,280	3,026
11	22	8	12	16
**12,617**	**12,213**	**11,886**	**11,279**	**10,616**
**49,720**	**49,269**	**46,586**	**41,775**	**39,175**
40,634	40,553	39,556	35,230	32,545
4,796	5,770	5,523	5,314	5,417
**6,064**	**7,335**	**7,058**	**6,834**	**7,073**
5,507	7,045	6,733	6,565	6,804
17	34	26	40	26
**55,824**	**56,615**	**53,649**	**48,612**	**46,249**
**2010**	**2011**	**2012**	**2013**	**2014**
**6,500**	**6,372**	**6,584**	**6,820**	**6,650**
5,223	5,402	5,651	5,888	5,681
647	697	736	758	818
**14,979**	**15,608**	**14,356**	**13,830**	**13,745**
13,653	14,849	13,775	13,384	13,391
35	78	36	25	34
**21,493**	**21,982**	**20,943**	**20,653**	**20,397**
**763**	**508**	**493**	**564**	**517**
570	415	427	497	452
114	68	51	56	58
**2,060**	**1,777**	**1,633**	**1,602**	**1,550**
1,860	1,699	1,567	1,558	1,513
2	7	5	2	2
**2,823**	**2,285**	**2,126**	**2,166**	**2,067**
**154**	**117**	**113**	**139**	**139**
74	66	60	76	67
65	45	46	59	68
**246**	**300**	**314**	**324**	**350**
217	277	295	311	338
0	0	1	2	0
**400**	**417**	**427**	**463**	**489**
**1,755**	**1,844**	**2,211**	**2,079**	**1,956**
962	1,150	1,374	1,277	1,125
558	598	745	727	772
**229**	**260**	**332**	**257**	**204**
202	240	315	235	199
0	0	4	2	1
**1,985**	**2,106**	**2,543**	**2,336**	**2,162**

For peer review only

**2,351**	**2,634**	**2,698**	**2,953**	**4,054**
472	478	470	491	480
1,618	2,036	2,129	2,375	3,477
**292**	**291**	**261**	**283**	**263**
259	250	239	255	243
3	6	8	7	2
**2,647**	**2,927**	**2,959**	**3,236**	**4,317**
**353**	**489**	**475**	**472**	**561**
269	400	445	439	524
37	44	7	9	11
**5,180**	**5,791**	**6,159**	**6,006**	**5,911**
4,279	5,069	5,828	5,676	5,626
18	15	11	17	20
**5,536**	**6,282**	**6,635**	**6,479**	**6,473**
**40,895**	**41,600**	**40,392**	**41,028**	**39,349**
34,011	36,134	35,632	36,535	34,611
2,657	3,004	3,120	3,156	3,456
**34,659**	**34,935**	**33,493**	**32,834**	**31,251**
30,968	32,672	31,929	31,601	30,055
122	228	148	173	152
**75,604**	**76,549**	**73,891**	**73,869**	**70,612**
**27,337**	**7,916**	**-**	**-**	**-**
21,042	6,795	-	-	-
3,277	962	-	-	-
**18,222**	**5,190**	**-**	**-**	**-**
16,266	5,095	-	-	-
51	26	-	-	-
**45,592**	**13,109**	**-**	**-**	**-**
**4,957**	**1,409**	**-**	**-**	**-**
2,448	737	-	-	-
1,921	628	-	-	-
**2,775**	**752**	**-**	**-**	**-**
2,432	735	-	-	-
8	1	-	-	-
**7,736**	**2,163**	**-**	**-**	**-**
**5,827**	**1,587**	**-**	**-**	**-**
4,546	1,347	-	-	-
728	222	-	-	-
**11,641**	**3,046**	**-**	**-**	**-**
10,546	3,004	-	-	-
13	7	-	-	-
**17,470**	**4,634**	**-**	**-**	**-**
**904**	**242**	**-**	**-**	**-**
173	40	-	-	-
617	191	-	-	-
**147**	**23**	**-**	**-**	**-**
121	22	-	-	-
0	0	-	-	-
**1,051**	**265**	**-**	**-**	**-**
**8,772**	**9,861**	**10,195**	**10,501**	**10,457**
7,023	8,137	8,655	8,965	8,853
788	1,111	1,125	1,204	1,314
**11,888**	**13,115**	**14,417**	**15,000**	**15,366**
10,515	12,164	13,781	14,536	14,908
36	80	41	42	62
**20,679**	**22,981**	**24,613**	**25,502**	**25,826**
**43,601**	**83,910**	**78,508**	**78,244**	**77,954**

For peer review only

13,692	26,989	28,199	26,685	25,143
24,646	52,754	47,640	47,790	50,021
**23,042**	**45,482**	**45,021**	**43,261**	**38,821**
20,988	41,825	42,986	40,623	37,209
89	698	148	130	138
**66,664**	**129,399**	**123,531**	**121,505**	**116,776**
**1**	**1**	**0**	**0**	**0**
-	-	-	-	-
-	-	-	-	-
**1**	**0**	**0**	**1**	**1**
-	-	-	-	-
-	-	-	-	-
**2**	**1**	**0**	**1**	**1**
**2010**	**2011**	**2012**	**2013**	**2014**
**0**	**0**	**0**	**0**	**0**
-	-	-	-	-
-	-	-	-	-
**0**	**0**	**0**	**0**	**0**
-	-	-	-	-
-	-	-	-	-
**0**	**0**	**0**	**0**	**0**
**0**	**2**	**4**	**0**	**4**
-	-	-	-	-
-	-	-	-	-
**3**	**2**	**3**	**3**	**11**
-	-	-	-	-
-	-	-	-	-
**3**	**4**	**7**	**3**	**15**
**1,066**	**1,187**	**1,173**	**1,260**	**1,220**
578	671	691	662	586
337	432	420	534	590
**677**	**693**	**653**	**650**	**637**
588	605	599	598	605
1	7	0	2	1
**1,748**	**1,880**	**1,826**	**1,912**	**1,858**
**36,201**	**38,803**	**39,685**	**41,124**	**41,409**
30,182	33,719	34,884	36,675	36,521
2,745	3,065	3,099	3,218	3,660
**20,853**	**22,431**	**21,847**	**22,057**	**21,794**
18,909	21,106	20,828	21,367	21,013
79	165	114	121	124
**57,090**	**61,254**	**61,536**	**63,182**	**63,208**
**9,166**	**9,739**	**9,867**	**9,224**	**9,423**
7,326	8,241	8,647	8,166	8,223
630	807	900	856	943
**72,183**	**77,727**	**80,217**	**78,790**	**77,374**
64,369	72,804	77,433	76,314	75,111
185	412	251	249	253
**81,422**	**87,477**	**90,087**	**88,019**	**86,804**
**-**	**-**	**-**	**-**	**-**
-	-	-	-	-
-	-	-	-	-
**1,285**	**1,638**	**1,696**	**1,944**	**1,688**
1,192	1,603	1,645	1,880	1,637
1	6	6	10	5
**1,285**	**1,638**	**1,696**	**1,944**	**1,688**
**-**	**-**	**-**	**-**	**-**

For peer review only

-	-	-	-	-
-	-	-	-	-
**204**	**226**	**277**	**502**	**515**
199	218	273	495	509
0	2	1	1	3
**204**	**226**	**277**	**502**	**515**
**4,559**	**1,070**	**-**	**-**	**-**
3,888	1,027	-	-	-
177	32	-	-	-
**6,271**	**1,769**	**-**	**-**	**-**
5,613	1,731	-	-	-
16	8	-	-	-
**10,835**	**2,840**	**-**	**-**	**-**
**-**	**13**	**12**	**4**	**80**
-	7	7	3	5
-	5	5	1	75
**-**	**6**	**4**	**3**	**4**
-	6	4	3	4
-	0	0	0	0
**-**	**19**	**16**	**7**	**84**
**794**	**974**	**946**	**885**	**920**
599	749	757	693	730
98	129	120	115	142
**381**	**504**	**489**	**474**	**471**
335	451	446	420	446
2	8	3	4	4
**1,176**	**1,479**	**1,435**	**1,359**	**1,391**
**754**	**828**	**711**	**742**	**722**
510	586	501	466	385
175	212	174	233	311
**249**	**267**	**378**	**271**	**265**
224	256	366	255	252
1	4	2	2	2
**1,003**	**1,095**	**1,089**	**1,013**	**987**
**87,725**	**103,129**	**118,027**	**121,144**	**125,185**
59,620	73,841	86,419	87,609	88,147
19,691	23,151	26,481	28,711	33,175
**76,035**	**94,333**	**115,236**	**122,011**	**127,539**
68,101	87,312	109,867	116,751	122,975
285	555	485	518	515
**163,864**	**197,489**	**233,270**	**243,183**	**252,743**
**2,527**	**2,526**	**2,468**	**2,360**	**2,287**
1,774	1,849	1,861	1,784	1,641
437	528	520	508	573
**14,601**	**15,904**	**15,518**	**15,064**	**15,580**
12,973	14,929	14,984	14,648	15,078
26	108	52	34	43
**17,143**	**18,432**	**17,986**	**17,426**	**17,867**
**14,656**	**17,501**	**17,519**	**16,441**	**15,545**
12,816	15,360	15,624	14,659	13,681
822	1,296	1,294	1,347	1,390
**97,665**	**107,018**	**107,118**	**103,839**	**100,636**
85,427	99,155	102,635	99,820	96,611
351	718	529	473	477
**112,445**	**124,548**	**124,644**	**120,295**	**116,206**
**2010**	**2011**	**2012**	**2013**	**2014**
**-**	**-**	**-**	**-**	**-**
-	-	-	-	-

For peer review only

-	-	-	-	-
**-**	**9,839**	**15,594**	**21,554**	**24,367**
-	9,328	14,830	20,919	23,505
-	73	111	140	134
**-**	**9,839**	**15,594**	**21,554**	**24,367**
**-**	**-**	**-**	**-**	**-**
-	-	-	-	-
-	-	-	-	-
**78,129**	**97,169**	**100,725**	**133,496**	**176,680**
70,237	89,957	96,363	129,326	167,566
167	335	265	233	293
**78,129**	**97,169**	**100,725**	**133,496**	**176,680**
**-**	**5,778**	**11,280**	**12,663**	**14,008**
-	2,168	4,196	4,957	5,431
-	3,427	6,606	7,325	8,068
**-**	**1,892**	**3,777**	**4,121**	**4,988**
-	1,766	3,484	3,846	4,683
-	15	48	49	50
**-**	**7,675**	**15,058**	**16,784**	**18,997**
**18,742**	**18,516**	**19,306**	**20,540**	**22,552**
6,655	6,884	7,170	7,649	8,483
9,911	10,595	11,219	12,148	13,409
**7,217**	**7,154**	**7,155**	**7,173**	**7,461**
6,192	6,478	6,535	6,687	7,080
103	141	111	102	92
**25,974**	**25,675**	**26,463**	**27,714**	**30,016**
**-**	**7,937**	**13,445**	**14,432**	**15,778**
-	2,870	4,809	5,376	5,741
-	4,495	7,884	8,597	9,594
**-**	**3,397**	**4,937**	**5,313**	**5,526**
-	3,018	4,481	4,974	5,243
-	62	72	74	59
**-**	**11,337**	**18,383**	**19,746**	**21,305**
**-**	**7,096**	**11,422**	**12,339**	**13,676**
-	2,461	4,195	4,480	4,874
-	4,199	6,678	7,488	8,439
**-**	**2,956**	**4,275**	**4,479**	**4,728**
-	2,646	3,899	4,192	4,511
-	58	59	65	38
**-**	**10,053**	**15,698**	**16,819**	**18,404**
**-**	**51**	**104**	**231**	**329**
-	29	27	29	36
-	21	77	195	276
**-**	**22**	**33**	**75**	**125**
-	21	33	74	118
-	0	0	0	2
**-**	**73**	**137**	**306**	**454**
**-**	**27**	**10**	**154**	**200**
-	12	6	9	16
-	15	3	145	181
**-**	**16**	**20**	**43**	**75**
-	14	20	42	71
-	1	0	0	2
**-**	**43**	**30**	**197**	**275**
**-**	**24**	**13**	**97**	**163**
-	10	2	6	18

For peer review only

-	14	10	90	145
**-**	**20**	**18**	**29**	**44**
-	20	18	28	43
-	0	0	0	0
**-**	**44**	**31**	**126**	**207**
**302,174**	**296,791**	**278,723**	**280,386**	**308,145**
214,452	221,849	203,784	199,539	206,964
44,408	54,504	60,410	66,717	88,204
**300,830**	**299,610**	**283,116**	**304,381**	**346,068**
260,040	278,465	267,437	287,937	325,680
1,129	1,687	1,644	1,613	1,480
**603,470**	**596,476**	**562,008**	**584,832**	**654,280**
**-**	**20,926**	**32,605**	**34,799**	**37,097**
-	17,069	27,647	29,727	31,003
-	2,158	3,558	3,886	4,914
**-**	**13,386**	**20,170**	**21,049**	**21,099**
-	12,228	19,235	20,299	20,332
-	122	77	87	63
**-**	**34,318**	**52,777**	**55,857**	**58,207**
**-**	**4,302**	**8,229**	**9,295**	**11,308**
-	1,638	3,037	3,223	3,671
-	2,335	4,898	5,778	7,295
**-**	**1,965**	**2,991**	**3,228**	**3,376**
-	1,755	2,817	3,070	3,238
-	10	25	33	13
**-**	**6,267**	**11,220**	**12,523**	**14,686**
**-**	**654**	**1,340**	**1,592**	**1,747**
-	218	441	540	571
-	415	877	1,018	1,150
**-**	**291**	**373**	**443**	**478**
-	275	362	421	466
-	1	4	4	4
**-**	**945**	**1,713**	**2,035**	**2,225**
**-**	**939**	**1,558**	**1,575**	**2,208**
-	226	346	336	353
-	678	1,166	1,193	1,814
**-**	**202**	**273**	**250**	**249**
-	183	257	233	245
-	7	6	5	2
**-**	**1,141**	**1,831**	**1,825**	**2,457**
**2010**	**2011**	**2012**	**2013**	**2014**
**-**	**3,190**	**4,844**	**5,365**	**7,133**
-	684	974	987	1,113
-	2,388	3,763	4,243	5,880
**-**	**800**	**1,018**	**1,049**	**1,237**
-	726	940	983	1,145
-	20	24	22	20
**-**	**3,991**	**5,863**	**6,414**	**8,371**
**1,185,889**	**1,078,555**	**1,146,031**	**1,098,929**	**1,090,425**
-	-	-	-	-
-	-	-	-	-
**1,760,361**	**1,704,900**	**2,474,840**	**2,440,094**	**2,448,643**
-	-	-	-	-
-	-	-	-	-
**2,955,508**	**2,789,919**	**3,654,722**	**3,560,707**	**3,558,841**
**556,929**	**589,115**	**608,728**	**635,065**	**668,801**
421,648	465,438	483,788	503,602	507,209
67,898	85,363	95,877	108,839	138,277

For peer review only

**609,808**	**660,751**	**680,712**	**726,830**	**767,284**
533,541	609,763	644,718	696,608	734,595
1,855	4,368	2,876	3,138	3,144
**1,167,718**	**1,250,115**	**1,289,585**	**1,362,095**	**1,436,339**
**628,960**	**489,440**	**537,303**	**463,864**	**421,624**
-	-	-	-	-
-	-	-	-	-
**1,150,553**	**1,044,149**	**1,794,128**	**1,713,264**	**1,681,359**
-	-	-	-	-
-	-	-	-	-
**1,787,790**	**1,539,804**	**2,365,137**	**2,198,612**	**2,122,502**
**-**	**4,146**	**4,130**	**3,107**	**1,329**
-	3,745	3,816	2,790	1,087
-	271	253	237	177
**-**	**3,957**	**4,014**	**3,925**	**2,400**
-	3,790	3,839	3,817	2,068
-	28	10	11	7
**-**	**8,104**	**8,144**	**7,032**	**3,730**
**463,091**	**494,849**	**518,735**	**543,475**	**567,928**
352,564	387,996	405,821	423,793	428,448
68,559	82,188	92,665	103,351	124,491
**463,091**	**503,685**	**522,689**	**551,743**	**575,756**
418,846	474,674	500,222	532,377	556,928
1,829	3,033	2,224	2,334	2,241
**926,999**	**998,724**	**1,041,527**	**1,095,386**	**1,143,899**
**29,762**	**36,687**	**42,010**	**43,529**	**40,511**
16,194	18,914	20,460	20,623	20,334
9,636	15,282	19,562	21,041	18,947
**20,275**	**24,487**	**26,261**	**27,166**	**28,434**
17,806	22,291	24,342	25,623	27,075
164	367	375	332	238
**50,056**	**61,182**	**68,278**	**70,707**	**68,954**
**112,328**	**104,850**	**101,469**	**106,058**	**113,859**
95,043	91,708	90,996	95,996	100,319
6,869	7,377	6,363	6,746	9,093
**161,776**	**159,105**	**160,562**	**194,435**	**249,017**
146,354	150,367	153,737	187,843	238,086
436	780	597	700	780
**274,181**	**263,992**	**262,056**	**300,518**	**362,924**
**-**	**22,469**	**39,807**	**46,405**	**64,584**
-	13,969	24,601	28,501	38,112
-	7,357	13,094	16,614	24,497
**-**	**26,789**	**48,189**	**61,260**	**93,534**
-	24,905	44,082	58,415	89,657
-	189	283	325	419
**-**	**49,271**	**88,157**	**107,677**	**158,136**
**2010**	**2011**	**2012**	**2013**	**2014**
**560,015**	**590,010**	**615,898**	**645,319**	**684,572**
423,735	464,902	487,149	508,985	515,094
68,645	86,593	99,351	113,410	145,863
**612,070**	**659,957**	**688,230**	**735,673**	**779,085**
535,663	608,991	651,929	705,121	746,307
1,870	4,373	2,894	3,169	3,181
**1,173,069**	**1,250,211**	**1,304,266**	**1,381,194**	**1,463,910**
**125,537**	**32,924**	**-**	**-**	**-**
95,640	27,379	-	-	-
16,706	4,779	-	-	-
**168,925**	**47,531**	**-**	**-**	**-**

150,491	46,376	-	-	-
459	188	-	-	-
**294,650**	**80,469**	**-**	**-**	**-**
**434,478**	**125,875**	**-**	**-**	**-**
328,095	106,530	-	-	-
51,939	17,009	-	-	-
**443,145**	**130,252**	**-**	**-**	**-**
385,172	127,222	-	-	-
1,411	636	-	-	-
**878,419**	**256,199**	**-**	**-**	**-**
**-**	**84,982**	**119,291**	**123,544**	**132,239**
-	67,374	96,445	98,950	99,890
-	10,761	17,326	20,242	27,201
**-**	**127,467**	**185,573**	**204,925**	**224,974**
-	115,813	175,996	196,717	214,745
-	773	707	779	811
**-**	**212,496**	**304,903**	**328,513**	**357,258**
**-**	**11,153**	**15,545**	**16,500**	**18,688**
-	3,536	4,191	3,928	3,889
-	7,107	10,946	12,270	14,499
**-**	**4,573**	**4,805**	**4,377**	**4,797**
-	4,136	4,584	4,189	4,633
-	68	46	55	37
**-**	**15,727**	**20,352**	**20,878**	**23,487**
**-**	**335,076**	**481,062**	**505,275**	**533,645**
-	260,083	386,513	406,107	411,315
-	46,937	71,079	80,898	104,163
**-**	**350,134**	**497,852**	**526,371**	**549,314**
-	315,444	471,349	504,215	526,929
-	2,708	2,141	2,335	2,333
**-**	**685,320**	**979,011**	**1,031,803**	**1,083,165**
**214,050**	**218,951**	**227,318**	**226,029**	**226,709**
142,427	150,436	150,198	148,908	143,244
23,866	31,551	35,092	39,257	48,206
**201,797**	**204,274**	**212,688**	**212,956**	**210,843**
121,093	134,397	132,464	135,489	136,737
365	726	465	510	448
**416,487**	**423,625**	**442,288**	**440,707**	**439,243**
**128,666**	**144,918**	**129,565**	**131,129**	**131,044**
79,684	78,435	72,429	72,987	71,103
35,059	59,365	52,284	52,746	55,585
**89,249**	**90,734**	**81,941**	**80,972**	**76,630**
80,365	84,556	78,194	77,124	73,735
277	984	303	295	325
**218,035**	**235,690**	**211,513**	**212,105**	**207,684**
**120,181**	**135,780**	**149,550**	**150,800**	**154,162**
86,533	101,660	113,816	113,380	112,812
22,030	26,160	29,494	31,771	36,609
**268,874**	**299,392**	**320,933**	**322,898**	**324,072**
238,433	278,465	307,653	310,586	312,623
867	1,821	1,329	1,291	1,302
**389,377**	**435,243**	**470,500**	**473,748**	**478,285**
**1,651,981**	**1,608,055**	**1,712,216**	**1,689,087**	**1,746,815**
756,079	847,188	879,460	908,796	937,789

139,469	198,697	239,166	269,546	339,878
**2,330,850**	**2,355,271**	**3,162,082**	**3,238,285**	**3,425,342**
1,036,292	1,215,458	1,295,156	1,460,004	1,662,436
3,869	8,254	6,557	6,915	6,827
**3,992,669**	**3,969,935**	**4,908,511**	**4,949,184**	**5,192,090**

ırable to data from previous years

For peer review only

BMJ Open

Analysis of the potential for point-of-care test to enable individualised treatment of infections caused by antimicrobial-resistant and susceptible strains of *Neisseria gonorrhoeae*

Journal:	*BMJ Open*
Manuscript ID	bmjopen-2016-015447.R1
Article Type:	Research
Date Submitted by the Author:	30-Mar-2017
Complete List of Authors:	Turner, Katy; Bristol University<br>Christensen, Hannah; University of Bristol, School of Social and Community Medicine<br>Adams, Elisabeth; Aquarius Population Health, Managing Director and Founder<br>McAdams, David ; Duke University, Duke Fuqua School of Business<br>Fifer, Helen; Public Health England Colindale, Bacteriology Reference Department<br>McDonnell, Anthony ; Wellcome Trust<br>Woodford, Neil; Public Health England, National Infection Service
**Primary Subject Heading**:	Infectious diseases
Secondary Subject Heading:	Diagnostics, Epidemiology, Sexual health
Keywords:	INFECTIOUS DISEASES, Diagnostic microbiology < INFECTIOUS DISEASES, HEALTH ECONOMICS, SEXUAL MEDICINE, BACTERIOLOGY

SCHOLARONE™
Manuscripts

For peer review only

Analysis of the potential for point-of-care test to enable individualised treatment of infections caused by antimicrobial-resistant and susceptible strains of *Neisseria gonorrhoeae*

Katy ME Turner, Hannah Christensen, Elisabeth J Adams, David McAdams, Helen Fifer, Anthony McDonnell, Neil Woodford

School of Veterinary Sciences, University of Bristol, Langford House, Langford, Bristol BS40 5DU, UK
Katy ME Turner
Senior Lecturer

School of Social and Community Medicine, University of Bristol, Oakfield House, Oakfield Grove, Bristol, BS8 2BN, UK
Hannah Christensen
Lecturer

Aquarius Population Health, 58a Highgate High Street, London N6 5HX, UK
Managing Director and Founder
Elisabeth Adams

Duke Fuqua School of Business, 100 Fuqua Drive, A416, Durham, NC 27708, USA
Professor of Business Administration and Economics
David McAdams

Bacteriology Reference Department, National Infection Service, Public Health England, London, UK
Consultant Microbiologist
Helen Fifer

The O'Neill Review on Antimicrobial Resistance, Wellcome Trust, London, UK
Head of Economic Research
Anthony McDonnell

Bacteriology Reference Department, National Infection Service, Public Health England, London, UK
Head, AMRHAI Reference Unit
and
The O'Neill Review on Antimicrobial Resistance, Wellcome Trust, London, UK
Scientific Advisor
Neil Woodford

Correspondence to:
Katy ME Turner
E: katy.turner@bristol.ac.uk
T: +44 (0) 117 3314563

Keywords: point-of-care test; Neisseria gonorrhoeae; antimicrobial-resistance

Word count: 3538
Excluding title page, abstract, references, figures and tables.

For peer review only

For peer review only - http://bmjopen.bmj.com/site/about/guidelines.xhtml

Abstract

Objective: To create a mathematical model to investigate the treatment impact and economic implications of introducing an antimicrobial resistance point-of-care test (AMR POCT) for gonorrhoea as a way of extending the life of current last-line treatments.

Design: Modelling study.

Setting: England.

Population: Patients accessing sexual health services.

Interventions: Incremental impact of introducing a hypothetical AMR POCT that could detect susceptibility to previous first line antibiotics e.g. ciprofloxacin or penicillin so that patients are given more tailored treatment, compared with the current situation where all patients are given therapy with ceftriaxone and azithromycin. The hypothetical intervention was assessed using a mathematical model developed in Excel. The model included initial and follow-up attendances, loss to follow-up, use of standard or tailored treatment, time taken to treatment and the costs of testing and treatment.

Main outcome measures: Number of doses of ceftriaxone saved, mean time to most appropriate treatment, mean number of visits per (infected) patient, number of patients lost to follow-up and total cost of testing.

Results: In the current situation an estimated 33,431 ceftriaxone treatments are administered annually and 792 gonococcal infections remain untreated due to loss to follow-up. The use of an AMR POCT for ciprofloxacin could reduce these ceftriaxone treatments by 66%, and for an AMR POCT for penicillin by 79%. The mean time for patients receiving an antibiotic treatment is reduced by 2 days in scenarios including POCT and no positive patients remain untreated through eliminating loss to follow-up. Such POCTs are estimated to add £34 million to testing costs, but this does not take into account reductions in costs of repeat attendances and the reuse of older, cheaper antimicrobials.

Conclusions: The introduction of AMR POCT could allow clinicians to discern between the majority of gonorrhoea-positive patients with strains that could be treated with older, previously abandoned first-line treatments, and those requiring our current last-line dual therapy. Such tests could extend the useful life of dual ceftriaxone and azithromycin therapy, thus pushing back the time when gonorrhoea may become untreatable.

For peer review only - http://bmjopen.bmj.com/site/about/guidelines.xhtml

Strengths and weaknesses

- This study uses a simple framework to evaluate the potential impact of point of care tests to diagnose antimicrobial resistant or sensitive gonorrhoea infections
- Parameterised with contemporary UK data on diagnoses, treatment and levels of antimicrobial resistance
- Uses a static model, so not possible to extrapolate future population effects

INTRODUCTION

Increasing antimicrobial resistant gonorrhoea represents a significant and urgent public health problem. Gonorrhoea, caused by *Neisseria gonorrhoeae* is the second most commonly diagnosed bacterial sexually transmitted infection (STI) in England. *N. gonorrhoeae* has evolved resistance to all major drug classes and has been recognised as a bacterium of international concern by World Health Organization (WHO) [1] and has been prioritised in the UK five-year antimicrobial resistance strategy[2].

Diagnoses have more than doubled from 16,839 in 2010 to 41,193 in 2015, mainly due to increased diagnoses in men who have sex with men (MSM), accounting for 70% of male infections in 2015, illustrated in Figure 1 [3] (data reported through GUMCADv2, including GUM clinics and other sexual health service providers, but not general practice). Infections are often asymptomatic, especially in women and in pharyngeal and rectal infections in MSM, but are still transmissible[4]. If untreated, complications of infection include pelvic inflammatory disease, infertility, increased risk of pregnancy complications and, in rare cases, life-threatening septicaemia[5]. Gonorrhoea infection also increases the risk of HIV acquisition[6].

[3]In the UK, the Gonococcal Resistance to Antimicrobial Surveillance Program (GRASP) has performed sentinel antibiotic susceptibility testing of gonorrhoea since 2000[7]. Increases in resistance to first line therapies resulted in two changes in treatment recommendation (Figure 1): from ciprofloxacin to cefixime in 2005 and then to ceftriaxone plus azithromycin in 2011 [7-9]. Our current first-line therapy is also our last-line option, and whilst the use of dual therapy is intended to delay resistance developing to ceftriaxone, decreased susceptibility to either of these drugs could lead to untreatable infections. Whilst new antibiotics are in development, their use in the clinic may be many years away and already the world's first reported clinical treatment failure with confirmed ceftriaxone and azithromycin resistance has occurred [7].

For peer review only

For peer review only - http://bmjopen.bmj.com/site/about/guidelines.xhtml

There are two main challenges to the management of gonorrhoea which contribute to the problem of resistance, illustrated in Figure 2. 1) Precautionary treatment: at the time of diagnosis, such that all infections are treated as if they are resistant to older antibiotics and 2) Epidemiological treatment: sexual contacts of gonorrhoea cases are often treated before diagnostic test results are known resulting in unnecessary treatment of uninfected partners. The cornerstone of gonorrhoea management to date has been to ensure rapid, highly effective treatment is given to prevent the onward spread of infection to sexual partners and to prevent people not returning for treatment following a diagnosis. In the context of antibiotic resistance and new diagnostic technologies, it is necessary to reassess these priorities.

Strategies are required to extend the life of existing antimicrobials for the successful treatment of gonorrhoea. Most infections diagnosed in the UK are susceptible to cefixime, ciprofloxacin and even penicillin [7]. Therefore, if a point-of-care test (POCT) could be developed to test for resistance (or susceptibility) to antibiotics, most patients could be treated with an older oral first-line therapy, potentially extending the life of ceftriaxone as our last-line therapy. [10]A promising option based on existing nucleic acid amplification test (NAAT) could be a PCR test for ciprofloxacin resistance, using the gyrA Gene as a target [10,11]. Other technologies could involve direct measurement of live cell responses to the presence of a panel of antibiotics including microfluidic devices, atomic force microscopy, volatile chemical detection or mass spectroscopy. Computational approaches based on *in silico* phenotyping based on genotype may also be able to detect new mutations more rapidly than traditional microbiological testing [12-14]. In this study we developed a mathematical model to investigate the treatment impact and economic implications of introducing an antimicrobial resistance (AMR) POCT for gonorrhoea.

METHODS

Model

We developed a decision tree model in Excel to consider the impact of a hypothetical new AMR POCT on testing, diagnosis and treatment of gonorrhoea in sexual health clinics in England (Figure 3), compared to current practice. Genitourinary clinics typically triage attending patients based on whether they have symptoms or report contact with a sexual partner infected with a specific infection (“same day management”) and those without symptoms (“delayed management”) where treatment is delayed until the results of diagnostic tests are returned from the laboratory (2-7 days) (Figure 2). Current practice is therefore a mixture of same day management and delayed management depending on clinic patient mix. Guidelines recommend that patients treated for gonorrhoea also have swabs taken at the time of treatment that are sent for susceptibility testing, but these results are not

For peer review only - http://bmjopen.bmj.com/site/about/guidelines.xhtml

available until after treatment has been given. The alternative strategy is based on a point of care gonorrhoea diagnostic test for all patients. The point of care test (POCT) could be either a simple diagnostic for gonorrhoea (infected/not infected) or a test which can discriminate between one specific resistance/susceptibility determinants (POCT AMR). Simple POCT tests are commercially available and have been piloted in clinic[15] but POCT AMR tests are still in development. More complex testing algorithms and diagnostic technologies could be envisioned, for example only using an AMR POCT if the initial simple POCT is positive (reflex testing) or using more complex algorithms and new technologies to determine optimal treatment options. In this preliminary example we consider two options of antimicrobial susceptibility 1) ciprofloxacin and 2) penicillin. [16,17]

The model was based upon an existing pathway model used to investigate the impact of introducing a dual POCT for gonorrhoea and chlamydia in a genitourinary medicine (GUM) setting[17,18], but simplified in that onward transmission of gonorrhoea and partner notification were not included, with the focus being on diagnosis and tailored treatment, shown in Figure 3 for MSM patient group (corresponding pathways for heterosexual men and women are given in the Appendix Figure A1 A-D). We explicitly included branches to differentiate susceptible and resistant isolates within the pathway framework. For the purpose of our study, we assumed that all point of care tests have equivalent sensitivity and specificity to current PCR laboratory tests. Previous models have considered variable specificity and sensitivity requirements in more detail [16].

Hypothetical cohorts of patients were followed through the pathway (MSM, heterosexual men and heterosexual women). Individuals could either receive same-day management or delayed management (Figure 3) under current practice or for POCT pathway all patients are assumed tested, diagnosed and treated on the same day. The only difference between POCT and AMR POCT is therefore in the choice of antimicrobial therapy. Treatments modelled were either our current last-line dual therapy of ceftriaxone and azithromycin (current pathway or simple POCT), or in the case of scenarios including AMR POCT a proportion of patients were provided with either ciprofloxacin or penicillin, plus azithromycin co-therapy, as an alternative regimen where possible. Loss to follow-up when patients were recalled for treatment following laboratory testing to determine positivity for gonorrhoea was explicitly included for current pathway only. We assumed that results of point of care diagnostics can be provided within the clinical consultation, e.g. if patients provide samples for testing on arrival at a GUM clinic and then wait for an appointment or return later in the day. It is possible that this would result in delays to treatment for symptomatic individuals and sexual contacts, but we do not consider this further.

For peer review only - http://bmjopen.bmj.com/site/about/guidelines.xhtml

Parameter values

Full model parameters are provided in the Appendix Table A1 and Table A2. Estimates of the numbers of patients attending GUM clinics and tested for and diagnosed with gonorrhoea were based on recent data from Public Health England (PHE)[19]. The model is run assuming 515,094 MSW, 145,863 MSM and 779,085 women attend a GUM clinic in 2014)[19] and the proportions entering same day management or who are infected adjusted to generate the observed diagnoses of gonorrhoea in each group. In 2014, there were over 33,000 diagnoses of gonorrhoea reported by PHE, just over half in men who have sex with men (MSM) and the remaining heterosexual cases split roughly equally between men and women. We combined data on patients presenting as contacts of gonorrhoea cases or with symptoms into the "same-day management" pathway. Asymptomatic patients were tested, but treatment was assumed to be delayed until the results of laboratory tests were known. We distributed infected patients between the pathways according to specific parameters for each patient group based on the probability of being infected and the likelihood of having symptoms. Symptomatic patients are more likely to be managed on the same day as testing and heterosexual men (MSW) are the most likely to be symptomatic, followed by MSM, then women. (Data from the Maximising STI Control trial, personal communication Cath Mercer) (Table 1) [17,18,20]. These parameters were informed by national PHE data where available and supplemented with additional data or clinical experience and are described fully elsewhere [17,20]. The difference between MSM and MSW may be due to a combination of factors including higher probability of extra-genital infection, higher incidence of repeat infections and higher probability of HIV coinfection and higher frequency of STI testing in this group.[21] We estimated the proportions of infections that are resistant to ciprofloxacin and/or penicillin from the GRASP 2014 report (Table A1), which included systematic susceptibility testing at the PHE reference laboratory from sentinel surveillance sites and a larger but less well defined analysis of samples tested locally[22]. Parameters were varied to be appropriate to three patient groups: heterosexual men, MSM, and women. In the baseline case we assumed that all confirmed and presumptive gonorrhoea infections are treated with ceftriaxone and azithromycin because there is >5% resistance to alternative regimens, resulting in 100% of infections treated as if they are resistant to other antibiotics (such as ciprofloxacin). The cost for patients attending GUM were taken from the latest payment by results tariff[23]. An AMR POCT is not currently available so we assumed conservatively that separate new tests for assessing resistance to either ciprofloxacin or penicillin would each incur an additional £25 testing cost, similar to that previously assumed for a PCR based POCT test[17]

For peer review only - http://bmjopen.bmj.com/site/about/guidelines.xhtml

Management scenarios

We considered the following scenarios for each of the three patient groups (MSM, heterosexual men and women).

1) Current management – clinicians have no knowledge of the resistance profile of gonorrhoea at the point of initial treatment and consequently all patients are treated with ceftriaxone and azithromycin. Some patients are managed on the same day, either due to symptoms and positive microscopy or as contacts of infected individuals, others wait for lab results, resulting in some unnecessary treatment and some delays to treatment or loss to follow-up. (Figure 2)
2) Simple POCT management – all patients tested and managed same day but all treated as if resistant to older antibiotics (i.e. ceftriaxone and azithromycin)
3) AMR POCT management - all patients tested with AMR POCT for gonorrhoea that could identify infections that do not need to be treated with ceftriaxone
   a. assuming current ciprofloxacin resistance prevalence[22]. (Figure 2)
   b. assuming current penicillin resistance prevalence[22].

Economic analysis

The primary outcomes were: the number of doses of ceftriaxone saved; and the mean time to appropriate treatment. In addition, we calculated the average number of visits per person and per infected person, the total cost of testing and the number of patients lost to follow up. In each case we compared the incremental benefit of an AMR POCT with current testing practice. Analyses were undertaken from the NHS perspective with costs measured in pounds sterling at 2014 prices.

RESULTS

We modelled a snapshot of GUM attendance, gonorrhoea diagnosis and prevalence of resistance to ciprofloxacin and penicillin based on the situation in England, 2014[19]. Under current treatment guidelines for 1.4 million people attending GUM per year we estimate 33,431 ceftriaxone treatments are currently administered annually and 792 gonococcal infections remain untreated due to loss to follow-up. In those receiving antibiotics, the mean time to treatment was estimated to be 2.2 days. Under current practice, 68% (MSW), 63% (MSM) and 21% (Women) who are infected with gonorrhoea are treated on the same day as they attend. The mean number of attendances at clinic per infected person was 1.44. We estimated the total cost of current testing to be £196 million. If a POCT test is used (strategies 2-4), this enables same-day testing and treatment, patients would only need to visit once, all infected individuals would be treated on the same day as the test and therefore no infected individuals would be lost to follow-up and left untreated.

For peer review only - http://bmjopen.bmj.com/site/about/guidelines.xhtml

The results for AMR POCT (strategy 3, 4) and POCT (strategy 2) only differ by the choice of treatment regimen. If an AMR POCT for ciprofloxacin resistance were available (strategy 3a) we estimate its use could prevent 22,054 treatments of ceftriaxone annually (a 66% reduction) assuming the current levels of resistance to ciprofloxacin (37% of infections in 2014[24],

For peer review only

For peer review only - http://bmjopen.bmj.com/site/about/guidelines.xhtml

Table 1). Similarly an AMR POCT for penicillin resistance (strategy 3b) at the current levels of resistance (23% overall) could prevent 26,499 ceftriaxone treatments annually (a 79% reduction). Assuming an AMR POCT added £25 to the testing costs we estimated the total cost of testing for each of the POCT scenarios to be £230 million, adding £34 million to the annual cost of testing.

DISCUSSION

Statement of principal findings

Our model estimates that 66% of the 33,431 ceftriaxone treatments given annually to individuals with gonorrhoea could be replaced by ciprofloxacin, thus extending the life of our current last-line treatment, if an AMR POCT for ciprofloxacin resistance was available. If an AMR POCT for penicillin was available, 79% of ceftriaxone treatments could be substituted with penicillin. The use of POCTs would mean a two day reduction in the time that people wait, on average, for appropriate treatment compared with current practice and such testing would prevent the approximately 800 positive individuals who remain untreated in the current system due to loss to follow-up. If AMR POCT added £25 to first-line testing costs, we estimate the use of such tests would increase current treatment and testing costs by £34 million annually. The outcomes related to same day diagnosis and treatment (reduced time to treatment and reduced follow up) could be achieved by using a simple POCT, as previously considered[17]. The additional benefit of AMR POCT test is to enable tailored choice of antimicrobial treatment.

Strengths and weaknesses of the study

Our model used recent published data on antimicrobial resistance levels, gonococcal incidence and current treatment and considered the impact of additional AMR POCT in distinct population groups, namely heterosexual men, MSM and females. The simplified model structure, which is available freely online, enables the parameters to be easily updated and the impact of different scenarios, in different settings, to be considered. We made the simplifying assumption that the cost of an AMR POCT would add £25 to the current tariff cost; however, in reality other current activities might be reduced or discontinued if an AMR POCT was available, such as testing, microscopy, culture and physical exams or re-attendances, as well as reduced costs associated with re-using cheaper oral antibiotics. New DNA-based POCT technologies may be able to be combined to produce a multiplexed test, which may be more economically viable than the separate specific AMR tests we modelled here. Our cost estimates are therefore likely to be higher than in practice. New technologies are emerging which may be able to rapidly determine the bacterial response to a panel of potential antibiotics which would enable highly tailored

For peer review only - http://bmjopen.bmj.com/site/about/guidelines.xhtml

therapy without the need to continuously monitor the efficacy of a test for resistance based on detecting DNA sequence, but for this preliminary exploration we selected a hypothetical AMR POCT test which could integrate with existing POCT technologies based on nucleic acid amplification.

The model did not capture the indirect effects of reduced transmission to partners or progression to complications, such as pelvic inflammatory disease and epididymitis. It also did not consider the longer term effects of changing treatment strategy on the evolution of drug resistance over time in gonorrhoea infections.

Strengths and weaknesses in relation to other studies, discussing important differences in results

To our knowledge, no-one has specifically addressed the question of the added value of a point-of-care AMR POCT to discriminate between susceptible and resistant strains to guide initial treatment decisions for gonorrhoea. Others have considered in detail the relative benefits of POCTs, balancing the need for fast results against cost and test performance[16]. Adams et al previously showed that a dual chlamydia/gonorrhoea point of care NAAT diagnostic test pathway could be cost neutral or cost-saving compared with existing methods even though the test kit itself is more expensive. [17,18] We initially assumed that the POCT AMR is an additional test cost, however it is probable that a multiplex PCR rapid test could be designed to include an AMR component which does not compromise the cost or performance of the basic gonorrhoea diagnostic. An alternative to improving diagnostics, treatment and surveillance is to develop a vaccine for gonorrhoea and to improve the uptake of other methods of prevention (such as condoms)[25,26]. A gonorrhoea vaccine has proved elusive due to the rapidly changing surface antigens, but there may be some cross-reactivity with vaccines designed to protect against *Neisseria meningitidis*[27].

The main weakness of our study is that it did not address the population level impact of the introduction of such tests, but only considered a static situation[24,25,28]. Rapid whole genome sequencing (within 24 hours) has been introduced to help guide treatment decisions for important nosocomial pathogens, notably MRSA (methicillin-resistant *Staphylococcus aureus*)[14], but in a community walk-in clinic setting for a low prevalence bacterial infection, such as gonorrhoea, a test needs to be relatively cheap and results available before the patient leaves the clinic . Our model did not include dynamic epidemiological or evolutionary processes, which change the prevalence and incidence of infection (and resistance) over time[24]. In reality, re-introduction of ciprofloxacin would likely increase the selection for resistance, which would negate some of the benefits of an AMR POCT. Similarly re-using

For peer review only - http://bmjopen.bmj.com/site/about/guidelines.xhtml

other drugs would also result in increases in resistance observed, including increasing selection for plasmids conferring multidrug resistance. Conversely, if point-of-care technology can reduce the time to treatment and reduce loss to follow-up sufficiently this might reduce the overall population prevalence, which would lead to a virtuous cycle of improved control and reduced transmission risk[29]. We also assume that results of point of care diagnostics can be provided within the clinical consultation. This is not currently possible unless the patient provides samples on arrival then waits to see a clinician or returns for a later appointment. The Cepheid GeneXpert has a turnaround time of about 90 minutes which was previously found to result in the majority of men (16/19) not waiting for their results (6 were positive)[30]. Transmission dynamic models can explore the potential consequences without the risks associated with radical changes in prescribing practices. The next steps will be to develop dynamic models which include selective pressure under differing treatment options[31] and incorporating variable delays.

The important next questions arising from this study are: how much time does the reduction in use of ceftriaxone buy in terms of slowing or preventing the emergence of clinically relevant gonorrhoea resistant to ceftriaxone and, second, what are the population-level benefits of improved gonorrhoea control?

Meaning of the study: possible explanations and implications for clinicians and policymakers

The major benefit of point of care tests for gonorrhoea is increasing the proportion of patients treated appropriately on the same day as the test, which is likely to improve outcomes by reducing infectious duration, reducing loss to follow-up and potentially improving partner notification efficacy. A definitive diagnosis on the day of first presentation also prevents unnecessary treatment of those not infected with gonorrhoea. The main benefit of an AMR POCT that can discriminate between susceptible and resistant infections is in enabling the re-introduction of abandoned first-line therapies. Reducing the use of antibiotics, especially of last-line therapies is a key aim of the UK national strategy on antimicrobial resistance. For heterosexual men and MSM a relatively large proportion of infections are already treated on the same day as testing, based on epidemiological, clinical or microbiological evidence (microscopy). However, this proportion is lower for women due to the higher percentage of asymptomatic infections and from poorer sensitivity of detection of gonorrhoea in endocervical and urethral smears. Although new POCTs are likely to be

For peer review only - http://bmjopen.bmj.com/site/about/guidelines.xhtml

more expensive than existing tests this would to some extent be offset by the reduction in further attendances and in the ability to re-use older, cheaper drugs. Given the low prevalence of gonorrhoea even in high-risk GUM attendees, the cost of treatment and re-attendances is small in comparison with the cost of attendances for testing and diagnosis. If a new discriminatory AMR POCT test were prohibitively expensive for routine use, a combination of a standard point-of-care NAAT (e.g. chlamydia/gonorrhoea) test could be considered in conjunction with a more specialised gonorrhoea AMR test, although the time implications of this for patients and clinicians would have to be carefully considered.

Unanswered questions and future research

This estimation of the potential reduction in ceftriaxone use is the first step towards evaluating the long-term effects of such a reduction. Future research investigating how much the useful lifespan of ceftriaxone as a therapy for gonorrhoea is extended with particular reductions in ceftriaxone use would be valuable. In the context of the often slow and expensive new drug pipeline, there is also a question to be answered around the value placed on each additional year of ceftriaxone availability.

For peer review only

For peer review only - http://bmjopen.bmj.com/site/about/guidelines.xhtml

Acknowledgments

We would like to acknowledge Lord Jim O'Neill, Chairman of the Review on Antimicrobial Resistance and the review team for commissioning the study.

Contributors

All authors were involved in the conception and design of the research. KT, EA and HC developed the models, following initial work by DM and NW and based on previous published work by EA & KT; KT and HC analysed the model results and all authors interpreted the results. HF and NW provided input into current clinical practice relating to AMR. KT, HC and NW wrote the first draft of the manuscript; all authors drafted the final version of the manuscript. All authors had full access to all of the data in the study and can take responsibility for the integrity of the data and the accuracy of the data analysis.

Transparency declaration

The lead author affirms that this manuscript is an honest, accurate, and transparent account of the study being reported; that no important aspects of the study have been omitted; and that any discrepancies from the study as planned have been explained.

Funding

The model was based on work previously funded by Aquarius Population Health[17,18]

KT, EA, KT and HC did not receive any funding from O'Neill review to undertake this project. HC was funded by the National Institute for Health Research Health Protection Research Unit (NIHR HPRU) in Evaluation of Interventions at the University of Bristol in partnership with Public Health England (PHE). The views expressed are those of the author(s) and not necessarily those of the NHS, the NIHR, the Department of Health or Public Health England.

Competing interests

KT reports grants from EPSRC, during the conduct of the study; personal fees from Aquarius Population Health, other from WHO, grants from Guys and St Thomas Charity , outside the submitted work; and I am an editor of Sexually Transmitted Infections .; HC reports grants from NIHR, during the conduct of the study; other from Sanofi Pasteur, outside the submitted work; EA reports no compensation for the submitted work, and grants from Cepheid, Atlas Genetics, St Georges University of London, Enigma Diagnostics, and AstraZeneca, outside the submitted work; AM reports personal fees from Department of Health, non-financial support from Wellcome Trust, outside the submitted work; NW reports PHE's AMRHAI Reference Unit receiving financial support from Achaogen Inc., Allecra Antiinfectives GmbH, Amplex, AstraZeneca UK Ltd, Becton Dickinson Diagnostics, BSAC,

For peer review only - http://bmjopen.bmj.com/site/about/guidelines.xhtml

Cepheid, Check-Points B.V., Cubist Pharmaceuticals, Department of Health, Enigma Diagnostics, Food Standards Agency, GlaxoSmithKline Services Ltd, Henry Stewart Talks, IHMA Ltd, Merck Sharpe & Dohme Corp., Meiji Seika Kiasya Ltd, Momentum Biosciences Ltd, Nordic Pharma Ltd, Norgine Pharmaceuticals, Rempex Pharmaceuticals Ltd, Rokitan Ltd, Smith & Nephew UK Ltd, Trius Therapeutics, VenatoRx and Wockhardt Ltd, outside the submitted work. DM and HF: no conflicts to declare.

Ethical approval

Ethical approval was not required for this research, which uses routinely collected data and data from other studies.

Data sharing

Details of the model data inputs and other assumptions are provided in the methods and supporting parameters table. The model is available from http://amr-review.org/file/429 and researchers interested in further details may contact the corresponding author at katy.turner@bristol.ac.uk

Copyright

The Corresponding Author has the right to grant on behalf of all authors and does grant on behalf of all authors, an exclusive licence on a worldwide basis to the BMJ Publishing Group Ltd to permit this article (if accepted) to be published in BMJ editions and any other BMJPGL products and sublicences such use and exploit all subsidiary rights, as set out in our licence.

For peer review only - http://bmjopen.bmj.com/site/about/guidelines.xhtml

Figure 1: Number of gonorrhoea diagnoses reported in England, 2006 – 2015, with the change in recommended first line antibiotic treatment shown.

Legend: Data from Public Health England, Annual STI Data Tables https://www.gov.uk/government/statistics/sexually-transmitted-infections-stis-annual-data-tables

Figure 2 Current patient pathways for gonorrhoea

Figure 3 Patient pathway diagram to illustrate the flow for men who have sex with men under A) current care, B) antimicrobial resistance point-of-care test

Legend: In scenario A, all diagnosed cases are treated with ceftriaxone plus azithromycin. In scenario B, diagnosed cases are treated according to resistance profile: AMR cases with ceftriaxone plus azithromycin; non-AMR with ciprofloxacin. Numbers of AMR and non-AMR infection are based on current levels of ciprofloxacin resistance observed in GRASP surveillance data, 2014. Illustrated based on 100,000 MSM attending a genitourinary medicine clinic.

For peer review only

For peer review only - http://bmjopen.bmj.com/site/about/guidelines.xhtml

Table 1 Principal results comparing use of an antimicrobial resistance point-of-care test (AMR POCT) for ciprofloxacin (Scenario 3a) or penicillin resistance (Scenario 3b) against current testing practice (standard laboratory testing, no POCT) for the management of gonorrhoea (Scenario 1), assuming the current attendance at GUM clinic annually

	Heterosexual male	MSM	Female	Overall
Considering use of POCT test for ciprofloxacin resistance				
Annual ceftriaxone treatments				
Current (scenario 1)	7690	17691	8050	33431
AMR POCT (scenario 3a)	2188	7933	1257	11378
Reduction under scenario 3a	5502	9759	6793	22054
Percentage reduction in ceftriaxone	72%	55%	84%	66%
Proportion treated same day				
Current (scenario 1)	68%	63%	21%	54%
AMR POCT (scenario 3a)	100%	100%	100%	100%
Increase under scenario 3a	32%	37%	79%	46%
Mean time to treatment (days)				
Current (scenario 1)	1.5	1.8	3.9	2.2
AMR POCT (scenario 3a)	0.0	0.0	0.0	0.0
Reduction under scenario 3a	1.5	1.8	3.9	2.2
Persons lost to follow up (untreated)				
Current (scenario 1)	125	338	329	792
AMR POCT (scenario 3a)	0	0	0	0
Considering use of POCT test for penicillin resistance				
Annual ceftriaxone treatments*				
Current (scenario 1)	7690	17691	8050	33431
AMR POCT (scenario 3b)	1407	4688	838	6932
Reduction under scenario 3b	6283	13004	7212	26499
Percentage reduction in ceftriaxone	82%	74%	90%	79%

*All other outcomes same as for use of POCT for ciprofloxacin resistance. MSM, men who have sex with men. Results for strategy 2 not shown – equivalent to strategy 3 except for choice of antibiotic treatment. Results for 3b also equivalent to 3a for outcomes except reduction in ceftriaxone treatments.

Definitions

GUM: Genitourinary medicine clinic, POCT: Point of care test, AMR: Antimicrobial resistance, MSM: Men who have sex with men

For peer review only - http://bmjopen.bmj.com/site/about/guidelines.xhtml

For peer review only

Table 2 Cost of testing and treatment* when using an antimicrobial resistance point-of-care test (AMR POCT) for ciprofloxacin resistance (strategy 3a) compared with current practice

	Heterosexual male	MSM	Female	Overall
Annual cost of testing				
Current	£69,784,517	£20,358,694	£105,826,467	£195,969,677
AMR POCT	£82,415,040	£23,338,080	£124,653,600	£230,406,720
Increased cost with AMR POCT	£12,630,523	£2,979,386	£18,827,133	£34,437,043

*The model assumes that the additional cost of AMR POCT (£25) is simply added to the cost of attendance, and is not offset by reductions in the number of gonorrhoea infections, by reduced treatment costs (as some patients are treated with cheaper antibiotics), or by reduced use of other tests (such as microscopy or culture of all swabs).

For peer review only

For peer review only - http://bmjopen.bmj.com/site/about/guidelines.xhtml

REFERENCE LIST

1. WHO. Antimicrobial resistance global report on surveillance: 2014 summary. . 2014.
2. Health UGDo. UK Five Year Antimicrobial Resistance Strategy 2013 to 2018., 2011.
3. Public Health England. STI diagnoses & rates by gender, sexual risk & age group, 2011 - 2015, 2016.
4. Korenromp EL, Sudaryo MK, de Vlas SJ, et al. What proportion of episodes of gonorrhoea and chlamydia becomes symptomatic? *Int J STD AIDS* 2002; **13**(2): 91-101.
5. Yeh JM, Hook EW, 3rd, Goldie SJ. A refined estimate of the average lifetime cost of pelvic inflammatory disease. *Sex Transm Dis* 2003; **30**(5): 369-78.
6. Chesson HW, Pinkerton SD. Sexually transmitted diseases and the increased risk for HIV transmission: implications for cost-effectiveness analyses of sexually transmitted disease prevention interventions. *J Acquir Immune Defic Syndr* 2000; **24**(1): 48-56.
7. England PH. GRASP Report 2016, 2016.
8. Bignell C, Fitzgerald M, Guideline Development G, British Association for Sexual H, Hiv UK. UK national guideline for the management of gonorrhoea in adults, 2011. *Int J STD AIDS* 2011; **22**(10): 541-7.
9. Public Health England GRASP Steering Committee and BASHH CEG. Gonorrhoea Treatment Position Statement 2015.
10. Hemarajata P, Yang S, Soge OO, Humphries RM, Klausner JD. Performance and Verification of a Real-Time PCR Assay Targeting the gyrA Gene for Prediction of Ciprofloxacin Resistance in Neisseria gonorrhoeae. *J Clin Microbiol* 2016; **54**(3): 805-8.
11. Chaudhry U, Ray K, Bala M, Saluja D. Mutation patterns in gyrA and parC genes of ciprofloxacin resistant isolates of Neisseria gonorrhoeae from India. *Sex Transm Infect* 2002; **78**(6): 440-4.
12. De Silva D, Peters J, Cole K, et al. Whole-genome sequencing to determine transmission of Neisseria gonorrhoeae: an observational study. *Lancet Infect Dis* 2016; **16**(11): 1295-303.
13. Allen VG, Melano RG. Whole-genome sequencing-new tools for gonorrhoea control. *Lancet Infect Dis* 2016; **16**(11): 1214-5.
14. Aanensen DM, Feil EJ, Holden MT, et al. Whole-Genome Sequencing for Routine Pathogen Surveillance in Public Health: a Population Snapshot of Invasive Staphylococcus aureus in Europe. *MBio* 2016; **7**(3).
15. Harding-Esch EM, Nori AV, Hegazi A, et al. Impact of deploying multiple point-of-care tests with a 'sample first' approach on a sexual health clinical care pathway. A service evaluation. *Sex Transm Infect* 2017.
16. Vickerman P, Watts C, Alary M, Mabey D, Peeling RW. Sensitivity requirements for the point of care diagnosis of Chlamydia trachomatis and Neisseria gonorrhoeae in women. *Sexually Transmitted Infections* 2003; **79**(5): 363-7.
17. Turner KM, Round J, Horner P, et al. An early evaluation of clinical and economic costs and benefits of implementing point of care NAAT tests for *Chlamydia trachomatis* and *Neisseria gonorrhoea* in genitourinary medicine clinics in England. *Sex Transm Infect* 2014; **90**(2): 104-11.
18. Adams EJ, Ehrlich A, Turner KM, et al. Mapping patient pathways and estimating resource use for point of care versus standard testing and treatment of chlamydia and gonorrhoea in genitourinary medicine clinics in the UK. *BMJ Open* 2014; **4**(7): e005322.
19. Public Health England. STI diagnoses & rates by gender, sexual risk & age group, 2010 - 2014, 2015.
20. Mercer CH, Macdonald N, Shirley MDF, et al. The Maximising STI Control (MSTIC) webtool: a new approach to facilitate the planning of services for sexually transmitted infections to maximise public health benefit. *Lancet* 2013; **382**: 6-.
21. Kent CK, Chaw JK, Wong W, et al. Prevalence of rectal, urethral, and pharyngeal chlamydia and gonorrhea detected in 2 clinical settings among men who have sex with men: San Francisco, California, 2003. *Clin Infect Dis* 2005; **41**(1): 67-74.

For peer review only - http://bmjopen.bmj.com/site/about/guidelines.xhtml

22. England. PH. Surveillance of antimicrobial resistance in *Neisseria gonorrhoeae*. Key findings from the 'Gonococcal resistance to antimicrobials surveillance programme' (GRASP) and related surveillance data, 2014.
23. Monitor and NHS England. 2014/15 National Tariff Payment System, 2013.
24. Grad YH, Goldstein E, Lipsitch M, White PJ. Improving Control of Antibiotic-Resistant Gonorrhea by Integrating Research Agendas Across Disciplines: Key Questions Arising From Mathematical Modeling. *J Infect Dis* 2016; **213**(6): 883-90.
25. Garnett GP. The theoretical impact and cost-effectiveness of vaccines that protect against sexually transmitted infections and disease. *Vaccine* 2014; **32**(14): 1536-42.
26. Regnier SA, Huels J. Potential impact of vaccination against Neisseria meningitidis on Neisseria gonorrhoeae in the United States: results from a decision-analysis model. *Hum Vaccin Immunother* 2014; **10**(12): 3737-45.
27. Whelan J, Klovstad H, Haugen IL, Holle MR, Storsaeter J. Ecologic Study of Meningococcal B Vaccine and Neisseria gonorrhoeae Infection, Norway. *Emerg Infect Dis* 2016; **22**(6): 1137-9.
28. O'Neill J. Tackling Drug-Resistant Infections Globally: final report and recommendations. The review on antimicrobial resistance, 2016.
29. White PJ, Ward H, Cassell JA, Mercer CH, Garnett GP. Vicious and virtuous circles in the dynamics of infectious disease and the provision of health care: gonorrhea in Britain as an example. *J Infect Dis* 2005; **192**(5): 824-36.
30. Harding-Esch EM, Hegazi A, Okolo O, et al. P2.163 Do "In-Clinic" Molecular and Non-Molecular Rapid Tests Improve Patient Management? *Sexually Transmitted Infections* 2013; **89**(Suppl 1): A137-A8.
31. McAdams D. Resistance diagnosis and the changing epidemiology of antibiotic resistance. *Ann N Y Acad Sci* 2017; **1388**(1): 5-17.

For peer review only

For peer review only - http://bmjopen.bmj.com/site/about/guidelines.xhtml

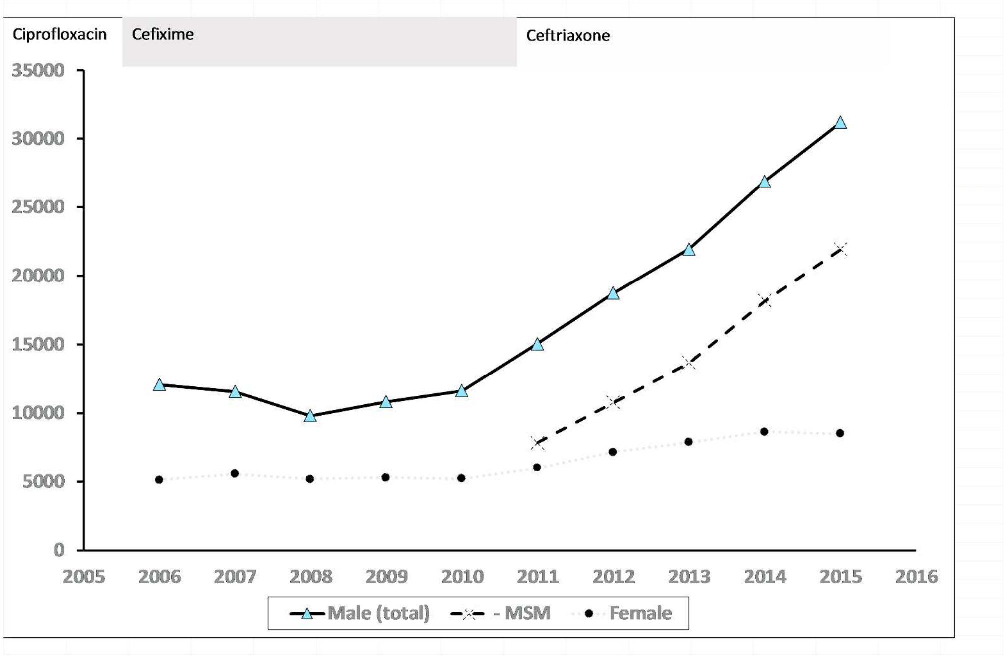

Figure 1

270x177mm (300 x 300 DPI)

For peer review only

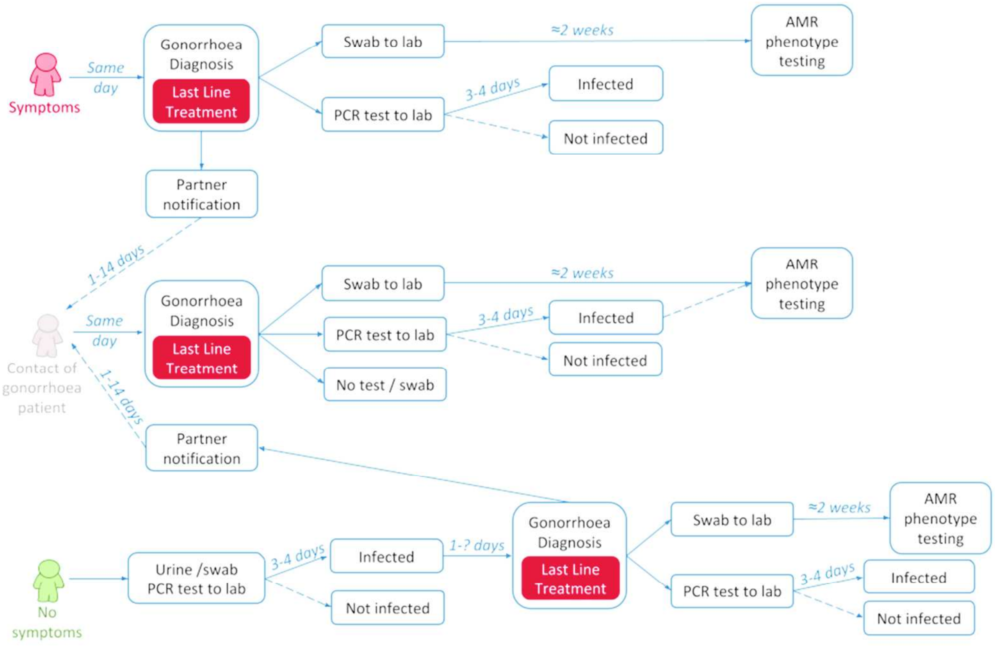

Figure 2

203x152mm (300 x 300 DPI)

For peer review only - http://bmjopen.bmj.com/site/about/guidelines.xhtml

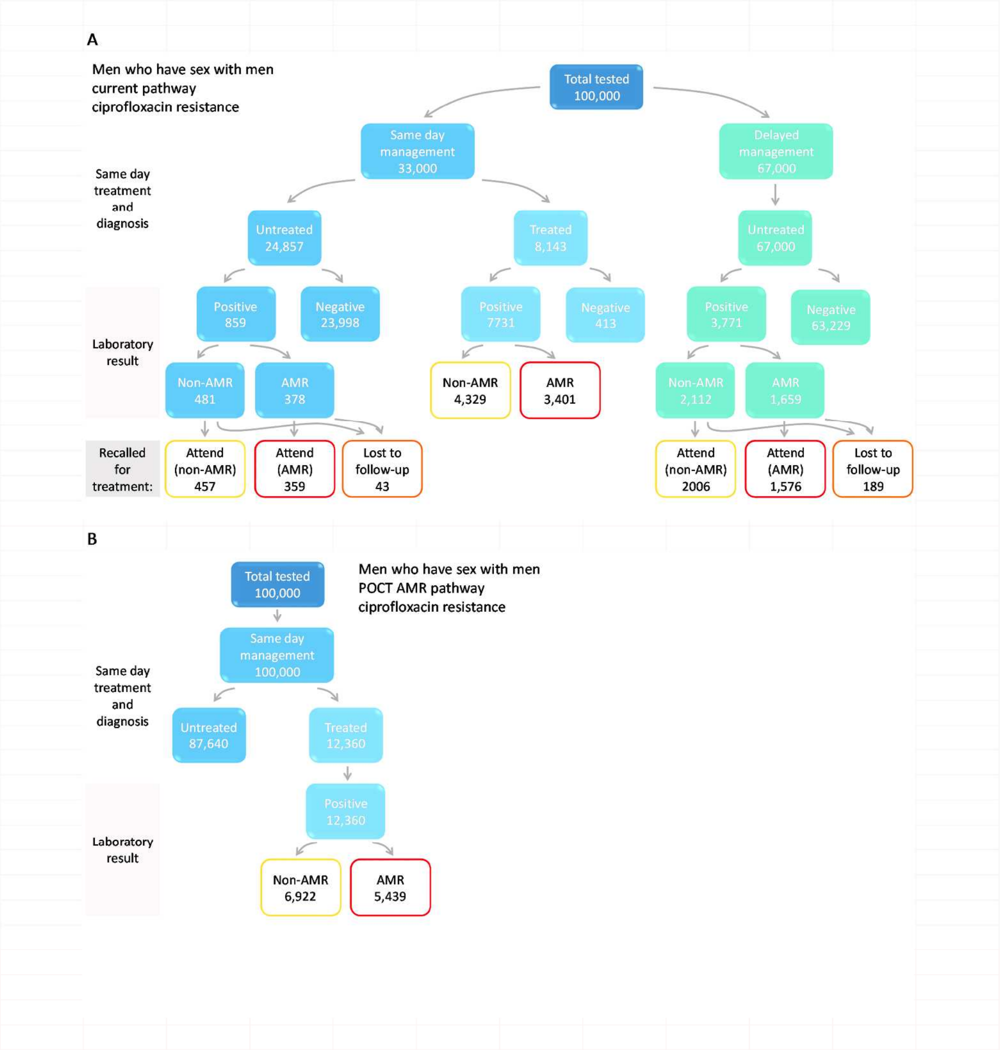

Figure 3

203x213mm (300 x 300 DPI)

DRAFT 29 June 2016

Supplementary information for:

Analysis of the potential for point-of-care test to enable individualised treatment of infections caused by antimicrobial-resistant and susceptible strains of *Neisseria gonorrhoeae*

Katy ME Turner, Hannah Christensen, Elisabeth J Adams, David McAdams, Helen Fifer, Anthony McDonnell, Neil Woodford

Table A1 Current prevalence of antimicrobial resistance to potential treatments for gonorrhoea

Drug	Class	Prevalence of resistance in GRASP 2014 isolates[1]			
		Heterosexual men	MSM	Women	Overall
Ceftriaxone	Cephalosporin (3rd generation)	0	0	0	0
Penicillin	β-lactam	18%	26%	10%	23%
Ciprofloxacin	Fluoroquinolone	28%	44%	15%	37%
Azithromycin	Macrolide	0.0%	1.4%	0.5%	1.0%

Current first line (and last-line) therapy is intramuscular ceftriaxone with azithromycin 1g orally. MSM, men who have sex with men.
GRASP: Gonococcal Resistance to Antimicrobial Surveillance Programme

For peer review only - http://bmjopen.bmj.com/site/about/guidelines.xhtml

Table A2 Model parameters

Baseline model parameters	Current			AMR POCT		
	Heterosexual men	MSM	Women	Heterosexual men	MSM	Women
Initial population size[2]	515,094	145,863	779,085	515,094	145,863	779,085
Proportion entering same day management pathway	35%	33%	48%	100%	100%	100%
Proportion infected with gonorrhoea (of total tested)[2]	1.5%	12.4%	1.1%	1.5%	12.4%	1.1%
Proportion of those in same day pathway infected with gonorrhoea	3.1%	26.0%	1.0%	1.5%	12.4%	1.1%
Proportion of delayed management infected with gonorrhoea	0.7%	5.6%	1.2%	-	-	-
Relative risk infection gonorrhoea in same day vs delayed pathway	4.52	4.63	0.82	-	-	-
Proportion in same day pathway who are infected & treated on same day	96%	90%	50%	100%	100%	100%
Proportion of same day pathway treated presumptively for gonorrhoea	5.0%	25.0%	2.0%	1.5%	12.4%	1.1%
Proportion who attend for treatment after lab test result (of those who wait for lab test results, i.e. asymptomatic group)	95%	95%	95%	100%	100%	100%
Proportion treated with last line therapy[3]	100%	100%	100%	28%[a]	44%[a]	15%[a]
Cost of first attendance[4 5]	£135	£135	£135	£135	£135	£135
Cost of follow-up attendance[4 5]	£104	£104	£104	£104	£104	£104

AMR POCT, antimicrobial resistance point of care test; MSM, men who have sex with men.

[a]Assuming POCT for ciprofloxacin susceptibility (can be adjusted for penicillin according to parameters in Table A1 or updated to reflect local trends)

For peer review only

For peer review only - http://bmjopen.bmj.com/site/about/guidelines.xhtml

1 **Figure A1**

2 **A – Heterosexual men current pathway**

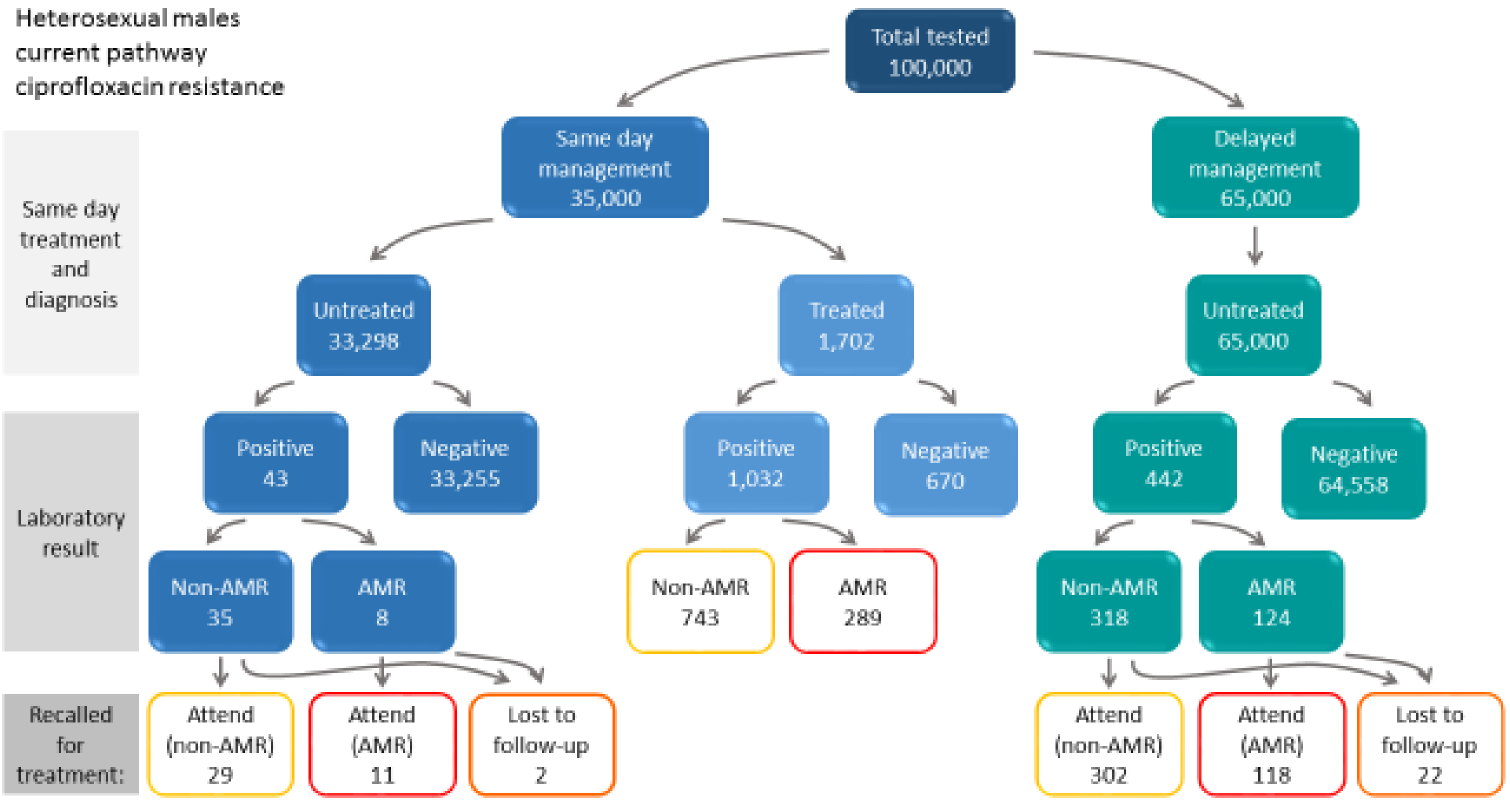

B – Heterosexual men POCT pathway

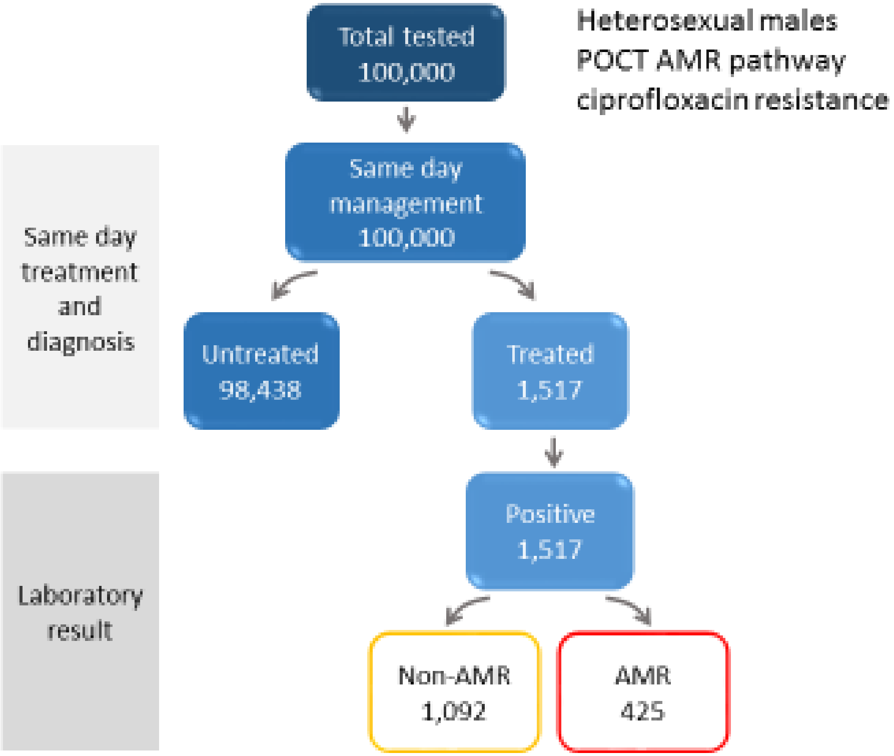

For peer review only - http://bmjopen.bmj.com/site/about/guidelines.xhtml

C – Women current pathway

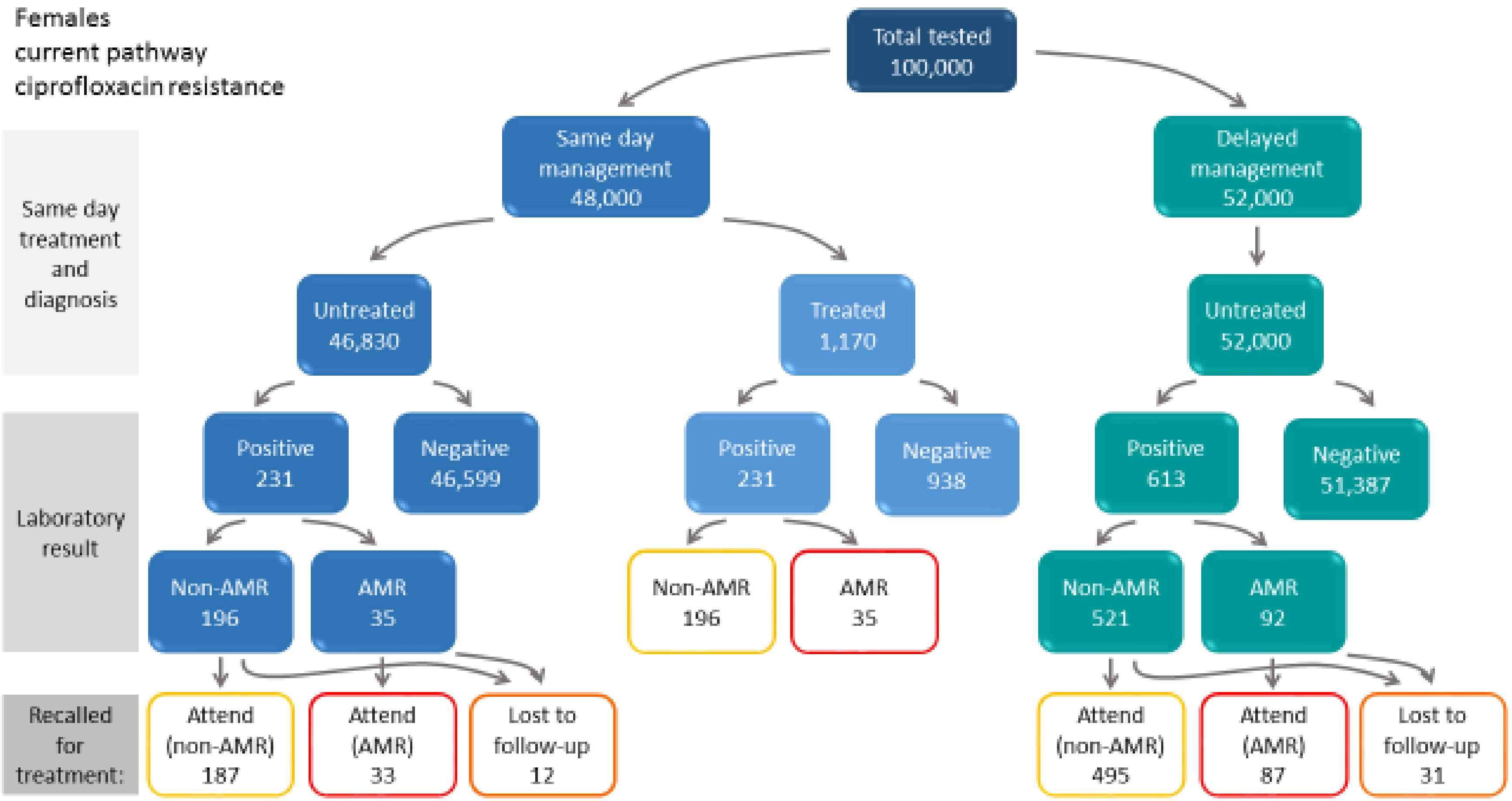

For peer review only - http://bmjopen.bmj.com/site/about/guidelines.xhtml

DRAFT 29 June 2016

1 **D – Women new pathway**

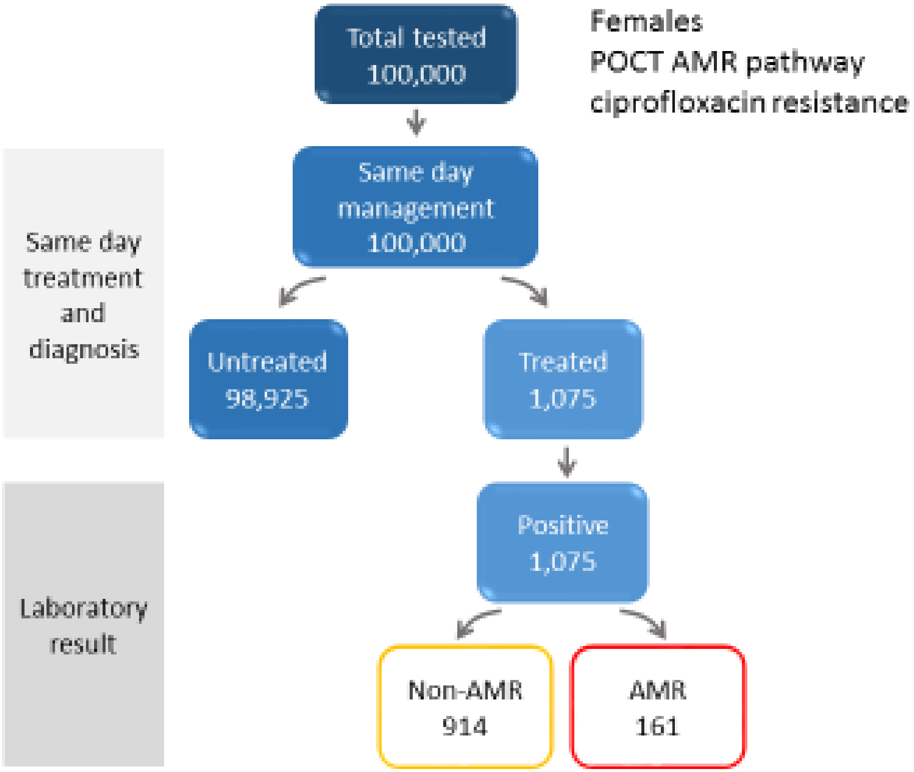

2

For peer review only - http://bmjopen.bmj.com/site/about/guidelines.xhtml

References

1 Adams EJ, Ehrlich A, Turner KM, Shah K, Macleod J, Goldenberg S, et al. Mapping patient pathways and estimating resource use for point of care versus standard testing and treatment of chlamydia and gonorrhoea in genitourinary medicine clinics in the UK. BMJ Open 2014; 4(7): e005322.

2 Public Health England. STI diagnoses & rates by gender, sexual risk & age group, 2010 - 2014, 2015.

3 England. PH. Surveillance of antimicrobial resistance in *Neisseria gonorrhoeae*. Key findings from the 'Gonococcal resistance to antimicrobials surveillance programme' (GRASP) and related surveillance data, 2014.

4 Monitor and NHS England. 2014/15 National Tariff Payment System, 2013.

5 Health PHEatDo. HIV, sexual and reproductive health: current issues bulletin2013. Available from: https://www.gov.uk/government/uploads/system/uploads/attachment_data/file/259087/HIV_Sexual_and_Reproductive_Health_bulletin-issue1nov2013.pdf.

6 Turner KM, Round J, Horner P, Macleod J, Goldenberg S, Deol A, et al. An early evaluation of clinical and economic costs and benefits of implementing point of care NAAT tests for *Chlamydia trachomatis* and *Neisseria gonorrhoea* in genitourinary medicine clinics in England. Sex Transm Infect 2014; 90(2): 104-11.

For peer review only

For peer review only - http://bmjopen.bmj.com/site/about/guidelines.xhtml
